# Updating perspectives on spinal cord function: motor coordination, timing, relational processing, and memory below the brain

**DOI:** 10.3389/fnsys.2024.1184597

**Published:** 2024-02-20

**Authors:** James W. Grau, Kelsey E. Hudson, David T. Johnston, Sienna R. Partipilo

**Affiliations:** Lab of Dr. James Grau, Department of Psychological and Brain Sciences, Cellular and Behavioral Neuroscience, Texas A&M University, College Station, TX, United States

**Keywords:** spinal cord injury, recovery, learning, pain, plasticity, metaplasticity, ionic plasticity

## Abstract

Those studying neural systems within the brain have historically assumed that lower-level processes in the spinal cord act in a mechanical manner, to relay afferent signals and execute motor commands. From this view, abstracting temporal and environmental relations is the province of the brain. Here we review work conducted over the last 50 years that challenges this perspective, demonstrating that mechanisms within the spinal cord can organize coordinated behavior (stepping), induce a lasting change in how pain (nociceptive) signals are processed, abstract stimulus–stimulus (Pavlovian) and response-outcome (instrumental) relations, and infer whether stimuli occur in a random or regular manner. The mechanisms that underlie these processes depend upon signal pathways (e.g., NMDA receptor mediated plasticity) analogous to those implicated in brain-dependent learning and memory. New data show that spinal cord injury (SCI) can enable plasticity within the spinal cord by reducing the inhibitory effect of GABA. It is suggested that the signals relayed to the brain may contain information about environmental relations and that spinal cord systems can coordinate action in response to descending signals from the brain. We further suggest that the study of stimulus processing, learning, memory, and cognitive-like processing in the spinal cord can inform our views of brain function, providing an attractive model system. Most importantly, the work has revealed new avenues of treatment for those that have suffered a SCI.

## Introduction

The study of the vertebrate central nervous system (CNS) has traditionally focused on the brain, with many adopting a systems approach wherein distinct functional capacities are linked to a particular neural structure. In this view, encoding spatial relations is ascribed to the hippocampus, executive function to the prefrontal cortex, and fear to the amygdala. Often implicit is a form of hierarchical control, wherein higher neural systems in the forebrain integrate sensory signals and organize motor commands that are relayed to lower-level processes in the brainstem and spinal cord, which are charged with faithfully executing the orders (Gallistel, 1980). In this scenario, the spinal cord functions as a conduit, relaying neural impulses to/from the brain, a capacity linked to the outer band of ascending/descending fibers (white matter). Little heed is paid to the inner region of the spinal cord (the central gray), which is seen as a kind of mechanical switchboard, driving ascending fibers and motoneurons in response to afferent sensory signals, modulated by descending fibers. While the central gray is recognized to have some capacity to organize simple (spinal) reflexes, such as withdrawal from a noxious stimulus, complex behavior, learning, and a sense of time are seen as the province of the brain.

Work by the lead author early in his career took a systems approach akin to that outlined above and characterized spinal cord mechanisms as operating in an unconditioned (unlearned) manner (Grau, 1987a,b; Meagher et al., 1989, 1990; McLemore et al., 1999; Crown et al., 2000). His trainees have systematically deconstructed this view, providing evidence that the spinal cord can learn, time, and integrate signals, yielding behavioral outcomes comparable to those taken as evidence of “cognition” in brain-dependent tasks (Allen et al., 2002; Grau, 2002; Grau et al., 2022). While these observations ran counter to prevailing views in psychology, they paralleled discoveries in the area of physiology, where researchers had recognized decades ago that neural circuits within the spinal cord can organize action and rhythmic behavior (Sherrington, 1906; Brown, 1914; Stuart and Hultborn, 2008). Building on this work, researchers showed that the isolated adult spinal cord could be trained to step and that the brain can induce a lasting change in behavior (a kind of memory) by modifying the action of a spinal circuit (Wolpaw and Lee, 1989; Edgerton et al., 1997; Patterson, 2001a). By the late 1990’s, a foundation had been laid, leading a group of us (J. W. Grau, M. M. Patterson, V. R. Edgerton, and J.R. Wolpaw) to organize a small conference to bring together the researchers who had questioned the traditional view of spinal cord function. The talks outlined the foundation for a revised view of spinal cord function, one that recognized the computational power of the spinal cord (Patterson, 2001b). From this view, the processing/integration of sensor signal and the execution of organized motor response is distributed across the nervous system, with local systems governing key functions, yielding a structure that is more heterarchical in nature (McCulloch, 1945; Cohen, 1992). In this paper, we will review these discoveries and provide an overview of what has been subsequently learned, referencing current reviews for additional details.

A key feature of the studies we will review is that the results do more than transform our view of CNS function—the results have clinical import, informing treatment for those who have suffered a spinal cord injury (SCI). The traditional view of the spinal cord suggested a bleak future for those with a SCI. If the system is hardwired, and has little capacity to organize behavior, an injury that cuts communication with the brain leaves little hope for recovery. If, in contrast, spinal cord systems can support key behavioral functions (e.g., stepping) with little input from the brain, discovering how to engage these systems offers some hope for recovery. Likewise, if systems within the spinal cord have some capacity for plasticity, this might be harnessed to encourage the adaptive rewiring of surviving circuits in response to neuronal growth and implants designed to span an injury.

In the sections that follow, we introduce key scientific discoveries and how these have impacted clinical treatment. The material is organized into sections, each of which highlights a particular set of findings, with a focus on those that challenge the traditional view of the spinal cord as an immutable relay of neural signals. While we will endeavor to highlight key findings, the scope of work conducted over the last five decades exceeds what can be reviewed here. For that reason, we will present a more personal perspective and refer the reader to other sources for additional details. We also recognize that readers will have varying backgrounds in key areas, with some having little knowledge of how the spinal cord is organized while others have less background on topics related to learning and memory. To address the former, we begin with an overview of the spinal cord and how it is organized. To address the latter, care is taken to unpack key concepts.

## Structure of the spinal cord

### Anatomy

**Figure 1:**
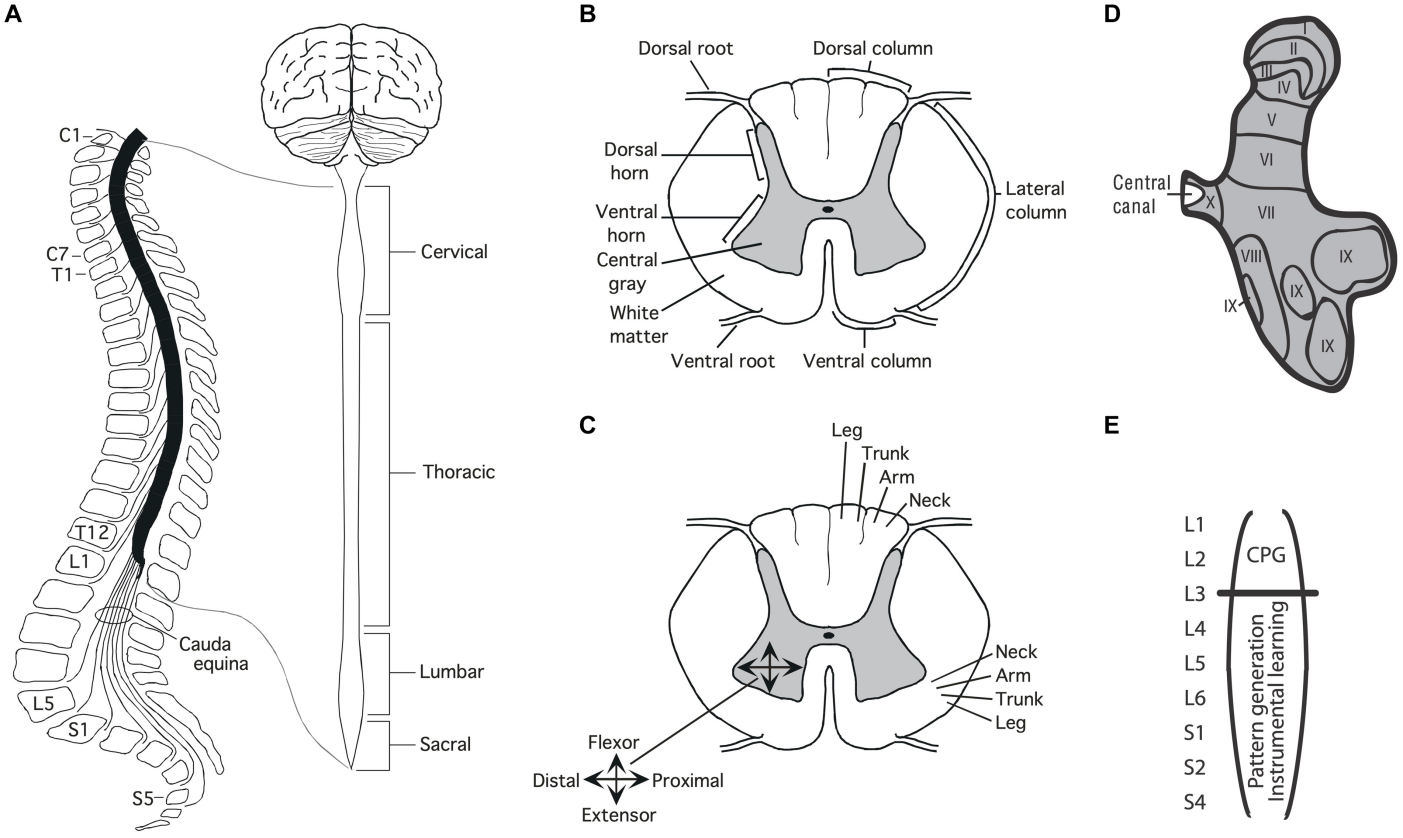
Anatomy of the spinal cord. **(A)** Gross anatomy of the spinal cord. Cross-sections of the spinal cord illustrating major structures **(B)**, functional organization **(C)**, and laminae **(D)** Adapted from Grau et al. (2022). **(E)** Research suggests that the central pattern generator (CPG) that drives the rhythm of stepping lies in the rostral lumbar region (L1-L2; Cazalets et al., 1995; Magnuson et al., 1999). The structures needed for instrumental learning, and that underlie the development of a learning deficit after uncontrollable stimulation, lie within the lower lumbosacral (L3-S2) spinal cord (Liu et al., 2005).

The soft tissue of the spinal cord is housed within a bony covering that is broken into segments (vertebrae) that are connected by fibrous tissue (ligaments), allowing for some flexibility (Figure 1A). Anatomists have divided the length of the spinal cord into 4 regions (Martin, 1996). The upper (rostral) region (immediately below the skull) is known as the cervical spinal cord, followed by the thoracic, lumbar and sacral. Within each region, the segments are numbered along the rostral to caudal (tail) axis. For example, C1-C7 for the cervical region and T1-T12 for the thoracic. Between the vertebrae, sensory nerves enter the spinal cord on the dorsal (toward the back) side while motor nerves exit from the ventral (toward the abdomen) side.

A cross-section of the spinal cord reveals two distinct regions: an outer ring of myelinated ascending/descending axons (white matter) and an inner region (gray matter) composed of cell bodies, dendrites, interneurons, and glia (Figure 1B). Unlike the brain, which is largely composed of projection neurons, the central gray is predominantly interneuronal, bolstering its integrative capacity (Hochman, 2007).

Regions of the central gray can be differentiated on the basis of the types of neural input they receive, their axonal projections, cell types, and function, yielding a laminae (layered) structure (Kirshblum et al., 2002). Laminae I to IV lie within the dorsal horn and receive input from cutaneous sensory neurons (Figure 1D). Laminae V-VII lie within the intermediate region; V and VI integrate proprioceptive signals related to movement and limb position, while laminae VII acts as a relay between the midbrain and cerebellum. Laminae VIII and IX lie in the ventral horn and coordinate/drive motor output. Additional subdivisions are suggested by research examining gene expression within the interneurons of the central gray (Jessell, 2000; Lee and Pfaff, 2001; Delile et al., 2019), which has revealed a myriad of distinct cell types that may subserve distinct functions.

### Development

Development brings an orderly distribution of fibers within the central gray (Figure 1C). For example, in the ventral region motor neurons innervating proximal muscles lie toward the medial (central) region while those deriving distal muscle groups are distributed in the lateral (side) ventral horn (Kirshblum et al., 2002; Grau et al., 2006). In addition, there is a division of labor across segments of the spinal cord. For example, neurons within the caudal (below L3) lumbosacral region coordinate the motor activity needed to generate lower-limb flexion/extension while neurons in the rostral lumbar (L1-L2) spinal cord contain a neural oscillator [a central pattern generator (CPG)] that drives rhythmic stepping behavior (Grillner and Zangger, 1979; Kiehn and Kjaerulff, 1998; Magnuson et al., 1999; Kiehn, 2006; Pocratsky et al., 2017).

**Figure 2:**
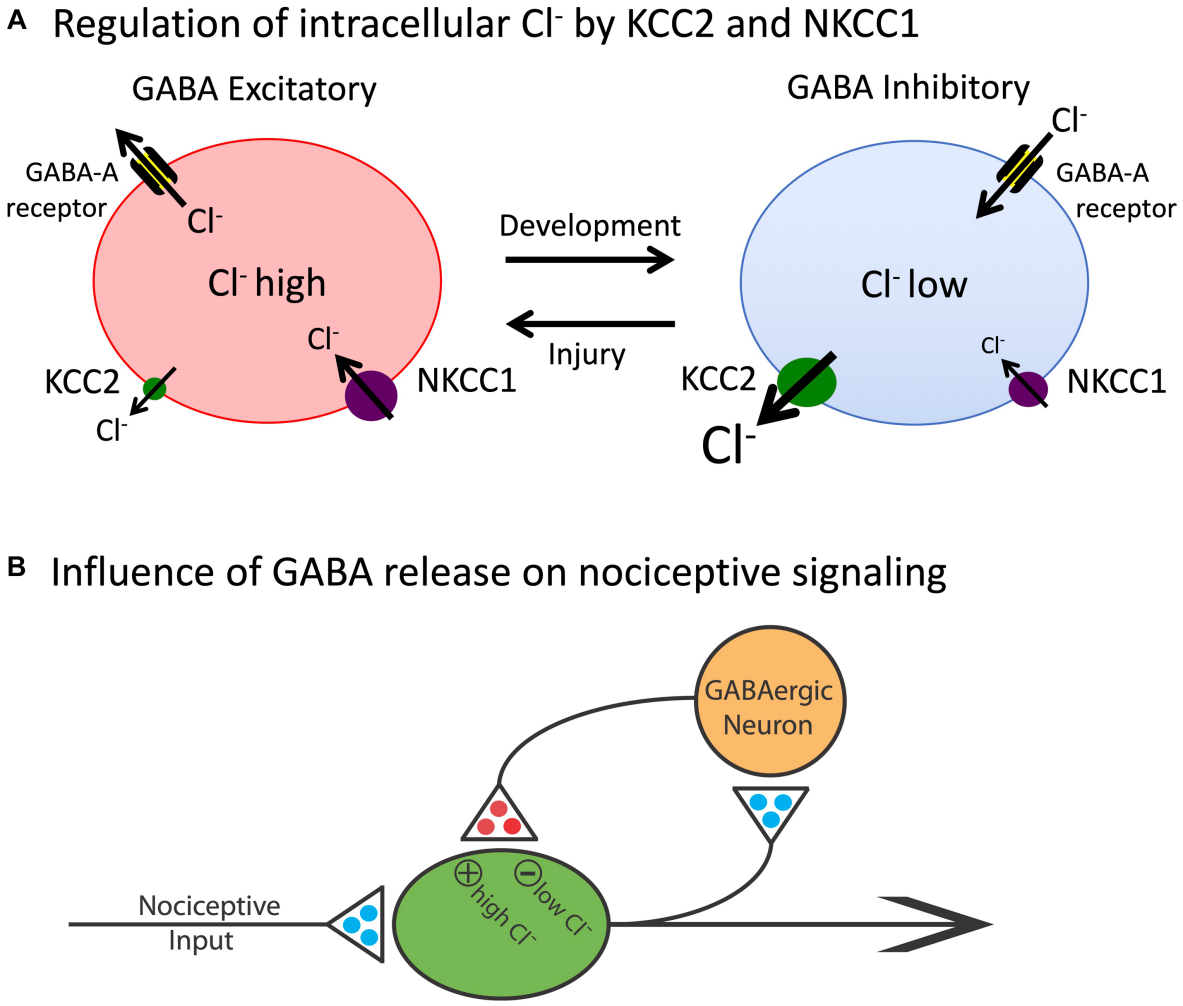
The release of GABA can have either an inhibitory (hyperpolarizing) or excitatory (depolarizing) effect depending upon the intracellular concentration of Cl^−^. **(A)** The co-transporters KCC2 and NKCC1 regulate the outward and inward flow of Cl^−^, respectively. In adult animals (right), the outward flow of Cl^−^ through the KCC2 channel maintains a low concentration of the anion within the cell. Under these conditions, engaging the GABA-A receptor allows Cl^−^ to enter the cell, which has a hyperpolarizing effect. Early in development, and after a rostral SCI, the levels of KCC2 are much lower and, as a consequence, there is a rise in the intracellular concentration of Cl^−^. Now, engaging the GABA-A receptor allows Cl^−^ to exit the cell, which has a depolarizing effect. **(B)** Nociceptive stimulation (input) will engage GABAergic neurons within the spinal cord that regulate neural excitability. In adult uninjured animals, the low intracellular concentration of Cl^−^ will cause GABA to have an inhibitory effect, which will dampen neural excitability. After injury, the reduction in membrane-bound KCC2 would transform how GABA release affects nociceptive circuits, causing it to have a depolarizing [excitatory (+)] effect that could contribute to the development of nociceptive sensitization and spasticity. Excitatory (glutamatergic) transmitters are indicated in blue and inhibitory (GABAergic) transmitters are colored red. Adapted from Grau et al. (2014).

The basic architecture of the spinal cord central gray is laid down early in development, encouraged by diminished gamma-aminobutyric acid (GABA) mediated inhibition (Ben-Ari, 2002, 2014). The neurotransmitter GABA primarily affects neural activity by engaging the ionotropic GABA-A receptor, which allows the anion Cl^−^ to cross the extracellular membrane (Figure 2). The direction of Cl^−^ flow depends upon its intracellular concentration, which is regulated by two co-transporters, KCC2 and NKCC1, that control the outward and inward flow of Cl^−^, respectively (Kaila et al., 2014; Medina et al., 2014). In the adult CNS, there is a high concentration of membrane-bound KCC2. This moves Cl^−^ out of the cell, which maintains a low intracellular concentration. Under these conditions, when the GABA-A receptor is engaged, Cl^−^ flows into the cell, producing a hyperpolarization that inhibits neural firing. But early in development, there is little KCC2 expression, which allows Cl^−^ to accumulate within the cell. Now, engaging the GABA-A receptor allows Cl^−^ to flow out of the cell, producing a depolarization that enhances neural excitability. It has been suggested that during early stages of development, this heightened excitation promotes the emergence synaptic circuits (Ben-Ari, 2002, 2014). Later in development, KCC2 expression is up-regulated, which dampens neural excitability, which could help preserve established neural circuits over time. The up-regulation of KCC2 has been linked to the maturation of descending fibers from the brainstem (Viemari et al., 2011).

Neurons within the white matter likewise develop in an orderly manner, laying down ascending/descending fiber tracts that serve distinct functions (Kirshblum et al., 2002). These fibers do more than relay signals to/from the brain; they also relay signals across distinct regions of the spinal cord. For example, the cervical and lumbar regions of the spinal cord are connected by propriospinal neurons that enable the coordination of fore/hind limb movement. Silencing these neurons disrupts left–right limb coupling/coordination (Pocratsky et al., 2020).

Early views of spinal cord function presumed that that axons within the white matter are hardwired in adults with little capacity to change, and unlike peripheral neurons, have little capacity for growth after injury (Patterson, 2001b). Research over the last 25 years has shown that this view is wrong on two counts. First, axons within the white matter demonstrate sprouting in adult animals and can re-innervate the central gray (Fouad et al., 2001; Vavrek et al., 2006). Second, while progress has been slow, researchers have shown that axonal growth can be fostered and produce functional re-innervation (Zheng and Tuszynski, 2023). These studies are complemented by work aiming to replace damaged neurons and glia, to rewire the spinal cord, re-establish the myelin sheath of surviving axons within the white matter, and use cell implants to replace lost tissue (Fischer et al., 2020).

### Peripheral innervation

Peripheral sensory signals are conducted by pseudounipolar neurons that have their cell bodies within the dorsal root ganglia (DRG), with a left/right pair at each vertebral level (Kirshblum et al., 2002). These neurons have two axon-like fibers, one of which projects to the periphery while the other innervates the spinal cord central gray. Myelinated (A) fibers carry signals tied to limb position (proprioception), touch, and fast pain. Unmyelinated (C) fibers transmit signals related to slow, burning, pain. A-fiber function can be further subdivided on the basis of its receptive ending. Sensory neurons can also be distinguished on the basis of chemicals that engage the fiber type. For example, a subset of pain (nociceptive) fibers express the transient receptor potential vanilloid 1 (TRPV1) receptor that is engaged by capsaicin (Willis, 2001), the active ingredient of chili peppers. Research exploring gene expression within sensory neurons has suggested additional subdivisions and provided the methodology needed to selectively engage or inhibit distinct fiber types (Iyer et al., 2016; Cowie et al., 2018; Takeoka and Arber, 2019; Guo et al., 2022; Kupari and Ernfors, 2023).

Skeletal muscles are innervated by motor neurons that have their cell bodies in the ventral horn, with axons that engage muscle contraction through the release of chemical transmitters at the neuromuscular junction (NMJ; Sanes and Lichtman, 2001). Traditionally, the primary transmitter at the NMJ in adult vertebrates has been assumed to be acetylcholine (ACh).

The peripheral nervous system (PNS) also regulates involuntary functions, such as heart rate, blood pressure, and digestion, by innervating smooth muscles and organs. Neurons from the parasympathetic NS, which fosters relaxation after periods of danger, include a number of cranial nerves and projections from the lower (sacral) region of the spinal cord (S2-4; Kirshblum et al., 2002). A state of arousal (fight-or-flight) is driven by the sympathetic component, which projects from the upper thoracic (T1) to the lumbar (L2-L3) segments of the spinal cord to ganglia that form a bilateral sympathetic chain that lies just ventral and lateral to the spinal cord tissue. These ganglia are inter-connected across segments, enabling coordinated output to peripheral processes. Surprisingly little is known about how signals within the sympathetic chain are coordinated or how they are affected by on-going processes (e.g., injury, inflammation).

### Injury

How a physical injury to the spinal cord affects function will depend upon its nature and locus. In the laboratory, researchers often cut (transect) the spinal cord in the upper thoracic (e.g., T2) region to elucidate what lower-level systems within the lumbosacral spinal cord can do minus communication with the brain (Grau et al., 2006). One consequence of a spinal transection is the loss of descending fibers that quell neural excitation, enabling the sensitization of nociceptive activity in the dorsal horn (Sandkuhler and Liu, 1998; Garraway and Hochman, 2001; Gjerstad et al., 2001; Huang et al., 2017; Grau and Huang, 2018). The loss also disrupts the regulation of sympathetic activity, which allows noxious stimulation to drive uncontrolled bouts of sympathetic activity, leading to accelerated heart rate, respiration, and sweating, a phenomenon known as autonomic dysreflexia (Krassioukov et al., 2003; Rabchevsky and Kitzman, 2011; Eldahan and Rabchevsky, 2018). Overtime, this uncontrolled activation of spinal sympathetic circuits worsens, which may be explained in part by the observation that the sympathetic circuity undergoes prolific axonal sprouting and plasticity after SCI (Noble et al., 2018).

While a large proportion of preclinical work is done using rats, key discoveries regarding the organization and function of motor systems have been made with a number of other species, including cats, zebrafish, and the lamprey (Cohen, 1992). In recent years, the development of mice that have distinct genetic anomalies, that enable researchers to selectively disrupt or express particular genes, has fueled the use of this species.

In humans, a complete transection is relatively uncommon, limited to injuries such as gunshot wounds. More commonly, the spinal cord is damaged by a deformation/bruising (contusion injury) that causes an acute tissue loss. The initial damage to the spinal cord initiates a pro-inflammatory cascade (list) that fosters additional tissue loss (secondary injury) over a period of hours to days (Crowe et al., 1997). Naturally, the effect of a contusion injury will depend upon both its severity and locus. A thoracic injury will lead to an insensitivity of stimuli below the waist and an accompanying motor paralysis of the lower limbs (paraplegia). Injuries in the cervical region will disrupt the ability to use the upper limbs, producing a tetraplegia (aka quadriplegia). Because a high-level tetraplegia will affect respiration, individuals may require a ventilator.

## Neurons in the spinal cord can coordinate complex behavior

Fifty years ago, most assumed that neural assemblies within the spinal cord can, at most, orchestrate simple reflexive behavior, such as a withdrawal from a noxious stimulus (Ladle et al., 2007). Beyond this, it was known that there was some coordination across limbs. For example, after a thoracic transection, flexion of one hind leg elicits an extension of the contralateral limb (crossed extension reflex; Sherrington, 1906). Likewise, it was known that rhythmic behavior could be elicited by the application of stimuli to the hind limbs in animals that had undergone a rostral transection (Sherrington, 1906). Further analysis showed that alternating flexor-extensor activity can occur without sensory input, implying the existence of a neural oscillator [central pattern generator (CPG)] within the spinal cord (Brown, 1914; Shurrager and Dykman, 1951; Lundberg, 1969). While research in this domain has traditionally characterized CPG activity in terms of a half-center model (Stuart and Hultborn, 2008; Cote et al., 2018), wherein rhythmic behavior is linked to excitatory/inhibitory pools of neurons linked in a reciprocal manner, recent data suggest an alternative view built upon a low-dimensional rotation of neural activity within the spinal cord (Linden et al., 2022).

It was initially assumed that the spinal CPG was a servant of the brain, which regulated its operation through descending fibers. Supporting this, researchers showed that coordinated stepping can be elicited by the local application of a drug (e.g., a noradrenergic agonist) that emulates neural activity in the descending pathway that drives locomotion (Forssberg and Grillner, 1973). From this perspective, while it was acknowledged that the spinal systems organized details of the muscular output, the brain served as the executor. It was within this climate that Rossignol, Edgerton, and their trainees, tried the seemingly impossible—to reestablish the capacity to step in adult spinally transected animals using behavioral training without drug therapy (Forssberg and Grillner, 1973; Grillner and Zangger, 1979; Forssberg et al., 1980; Smith et al., 1983; Edgerton et al., 1992; Hodgson et al., 1994; de Leon and Dy, 2017). Each day, spinally transected cats had their hindlimbs positioned on a treadmill while the upper body was supported. Of course, little hindlimb movement was observed at first, with the hindlegs dragged behind as the treadmill moved beneath. Yet, with some behavioral support (e.g., lifting the hind quarters and/or stimulating the perineum) and weeks of training, the animals slowly recovered the capacity to step. Further, as stepping returned, it adjusted to variation in treadmill speed. Minus input from the brain, or pharmacological intervention, neural systems within the spinal cord could be trained to step. It is presumed here that this training did not “teach” the animals to perform coordinated stepping, but instead reawakened a dormant circuit in the lumbosacral spinal cord.

An interesting feature of the spinal locomotor system is that it can gate afferent stimulation on the basis of step cycle. If the dorsal surface of the hind paw encounters an obstacle as the leg is lifted (swing phase), a flexion is elicited; if the same stimulus is applied while the leg moves rearward (extension), the leg is extended (Forssberg et al., 1977; Forssberg, 1979). And if an obstacle is repeatedly encountered at the same place during the swing phase, the magnitude of the flexion response gets stronger over trials and this effect remains for a number of steps after the obstacle is removed, suggesting a kind of learning (Edgerton et al., 1997, 2004). Such coordinated stepping requires: (1) a CPG with an adjustable frequency; (2) a pattern-formation network to shape the excitatory/inhibitory signals; and (3) the capacity to adapt to a changing environment (Windhorst, 2007). The spinal cord is not a simple reflexive machine.

Subsequent work built on these observations with the hope of fostering the recovery of locomotor performance (de Leon and Dy, 2017). Researchers found that the application of brain-derived neurotrophic factor (BDNF) or serotonin (5HT) to the lumbosacral spinal cord enhances stepping behavior (Rossignol et al., 1998; Lopez-Garcia, 2006; Boyce and Mendell, 2014). So too does electrical stimulation applied to the dorsal (epidural) surface of the spinal cord, an effect that seems related to the activation of afferent neural activity (Harkema et al., 2011; Angeli et al., 2014; Harkema et al., 2022). Remarkably, epidural stimulation can also enable voluntary movement in humans.

More recent work has revealed that step training can have a therapeutic effect that impacts other pathologies too, for example, counter chronic pain (Cote et al., 2014; Detloff et al., 2014; Tashiro et al., 2015). The benefit of training and exercise has been linked to increased expression of KCC2, which helps re-establish GABA-dependent inhibition. In the dorsal horn, this can counter the sensitization of pain (nociceptive) pathways that drive chronic pain (Huang et al., 2016). In the ventral horn, enhanced inhibition can reduce the over-excitation of motor circuits (spasticity) that often emerges after SCI, which could enable locomotor performance (Boulenguez et al., 2010).

Training can also promote the adaptive rewiring of spinal circuits. A particularly interesting example of this is provided by a paradigm wherein animals receive bilateral hemisections at different regions of the thoracic spinal cord. Each hemisection cuts all ascending/descending fibers for one side of the body; together, all fibers are cut. What is of interest is that an interneuronal bridge can form between the spared fibers from opposite sides, restoring communication across the injury, bringing some recovery of sensory/motor function (Courtine et al., 2008; Courtine and Sofroniew, 2019). The development of this neuronal bridge is encouraged by locomotor training and treatments that help restore GABA-dependent inhibition (Chen et al., 2018).

## Brain systems can induce a lasting modification in spinal cord function

It has been known for decades that brain systems can modulate spinal reflexes through descending tracts. This effect can be studied in the laboratory using an electrical analog of the stretch reflex—the Hoffman reflex (H-reflex). Wolpaw and his colleagues trained monkeys to alter the magnitude of the H-reflex by rewarding animals with fruit juice for exhibiting a change (e.g., a stronger) in reflex magnitude (Wolpaw and Lee, 1989; Wolpaw and Carp, 1990). Here, brain-dependent processes encode that there is a relationship between the behavioral response (e.g., exhibiting a stronger H-reflex) and the outcome (fruit juice), a kind of learning known as instrumental conditioning (aka operant learning). With training, they found that animals exhibited a change in H-reflex amplitude, demonstrating regulation of the spinal reflex by brain processes. After extended training, Wolpaw transected the spinal cord rostral to the region that mediates the reflexive response. Remarkably, the alteration in H-reflex magnitude remained, implying that brain-dependent processes can bring about a lasting alteration (memory) in the spinal cord. Interesting, how this spinal memory is laid down appears to depend more on the duration of conditioning than on the number of training trials (Wolpaw, 2018), implying that the modification that underlies the modification of the spinal circuit involves a kind of time-dependent consolidation.

**Figure 3:**
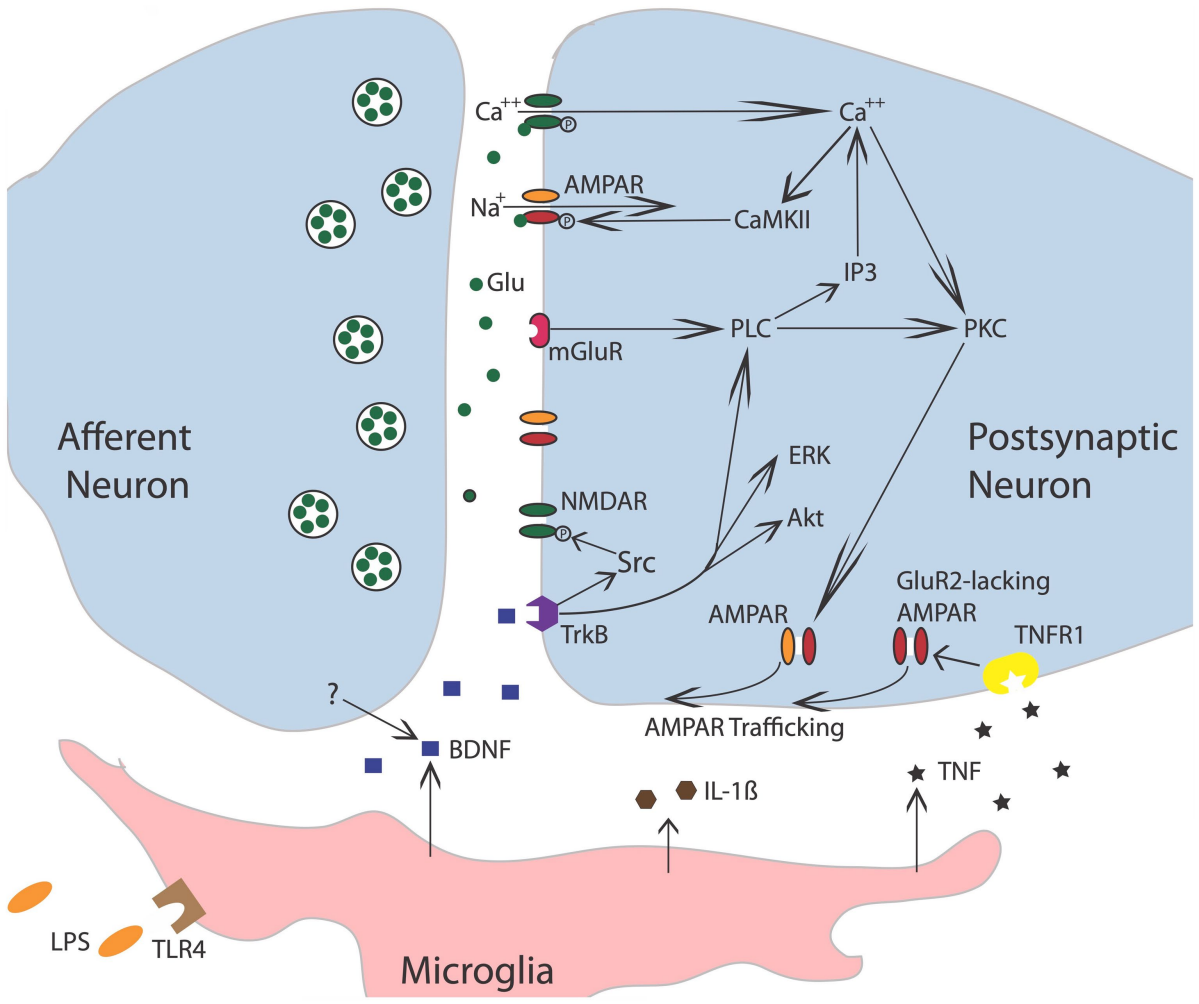
Nociceptive stimulation engages neurons within the spinal cord that release the neurotransmitter glutamate (Glu), engaging signal pathways implicated in plasticity. Akt, protein kinase B; AMPAR, a-amino-3-hydroxy-5-methyl-4-isoxazolepropionic acid receptor; BDNF, brain-derived neurotrophic factor; CaMKII, calcium/calmodulin activated protein kinase II; ERK, extracellular signal-regulated kinase; GluR2, glutamate receptor 2; IL-1b, interleukin-1 beta; IP3, inositol 1,4,5-trisphosphate; mGluR, metabotropic glutamate receptor; NMDAR, N-methyl-D-aspartate receptor; PKC, protein kinase C; PLC, phospholipase C; TrkB, tropomyosin receptor kinase B; TNF, tumor necrosis factor; TNFR1, TNF receptor 1. Adapted from Grau et al. (2014).

Further evidence that brain systems can induce a lasting modification in spinal cord function comes from work examining the phenomenon of spinal fixation. This was first described by DiGiorgio (1929), who showed that a cerebellar lesion produced a hindlimb postural asymmetry, involving the flexion of one limb and the extension of the other, in anesthetized animals. More interestingly, this brain-injury-induced asymmetry remained after the spinal cord was transected. It was naturally hypothesized that the cerebellar damage induces an alteration in the spinal circuitry through descending fibers. Like other examples of memory, the development of spinal fixation was disrupted by drug treatments that block the NMDA receptor (NMDAR) or protein synthesis (Patterson, 2001b). The NMDA receptor is of interest to those studying learning and memory because activating it requires both presynaptic transmitter release and a strong postsynaptic depolarization (Bliss and Collingridge, 1993; Morris, 2013), providing a form of coincidence detection (a Hebbian synapse). Engaging the NMDAR allows Ca^++^ to flow into the cell, which activates signal pathways that amplify the post-synaptic response to transmitter (glutamate) release (e.g., by trafficking AMPA receptors to the active zone of the synapse; Figure 3). Given many well-studied forms of brain-dependent learning and memory depend upon NMDAR-mediated plasticity, evidence that pretreatment with a NMDAR antagonist blocks the development of spinal fixation suggested a commonality in signal pathways and function—that neurons within the spinal cord are plastic and that this process depends upon neurochemical mechanisms analogous to identified within the brain.

Subsequent work has implicated peripheral processes in the induction of spinal fixation. The first evidence for this came from studies examining the potential role of endogenous opioids. Systemic treatment with drugs that engage the kappa or mu opioid receptor induce a lasting flexion in the left hind leg while administration of a delta opioid agonist produce flexion on the right side (Chazov et al., 1981; Bakalkin, 1989; Bakalkin and Kobylyansky, 1989). Perhaps most surprising, Lukoyanov and colleagues showed that a unilateral brain injury can induce postural asymmetry even when it is preceded by a spinal transection, implying that the alteration in motor behavior does not necessarily depend upon descending fibers (Lukoyanov et al., 2021). They posited that the brain may be impacting spinal cord function by means of a blood borne factor. To explore this possibility, they induced a unilateral brain injury in rats and then collected the animal’s serum. When this serum was administered to uninjured rats, it induced a comparable postural asymmetry. An even more remarkable outcome was obtained when pregnant dams were given a unilateral brain injury. Offspring from injured rats exhibited postural asymmetry and this effect too survived a spinal transection (Carvalho et al., 2021). These spinally-mediated alterations have been linked to distinct patterns of gene expression within the spinal cord.

The recognition that spinal circuits are inherently plastic raises a computational problem, because many systems may share a structural component. The hierarchical view of CNS function gains simplicity by assuming lower-level components function in a mechanical manner, assuring that execution of a command reliably elicits a particular motor response. Flexibility in this system was attributed to executive systems within the most rostral regions of the forebrain (e.g., prefrontal cortex). Recognizing that lower-level processes are plastic raises two inter-related problems. First, the higher-level system that evoked the modification would need to adjust the output, to compensate for variation in the vigor of the response elicited by a descending signal. To address this issue, Wolpaw has suggested that behavioral systems seek a form of *negotiated equilibrium* (Wolpaw, 2018). A similar view was suggested by Turvey, who proposed that higher processes “enter into ‘negotiations’ with lower domains in order to determine how the higher representation [of an action] shall be stated” (Turvey, 1977; Gallistel, 1980).

The second and more thorny issue stems from the way in which complex behavior is often assembled, with multiple systems sharing common components. Within such a system, a modification that profits the execution of one behavioral process would impact multiple systems, potentially causing a maladaptive consequence. This challenge, together with the recognition that “lower-level” processes may often have considerable computational power, has led some to propose that behavioral processes such as locomotion have an organizational structure that is better described as heterarchical, wherein “each level of the system contributes to the output, and each level helps to shape the final output of the system, and each is shaped in turn by the others” (Cohen, 1992). Here, the structure involves more of a *relative hierarchy* (Gallistel, 1980), wherein the ranking of units is labile rather than fixed, with the order of subordination context dependent.

Building on these views, Wolpaw has suggested the concept of a *heksor*, which he defines as “widely distributed network of neurons and synapses that produces an adaptive behaviour and changes itself as needed in order to maintain the key features of the behaviour” (Wolpaw and Kamesar, 2022). Such a view appears broadly consistent with the behavior systems approach, which is designed to address the flexibility of motivated behavior (Timberlake and Lucas, 1989; Timberlake, 1990; Grau and Joynes, 2001). Timberlake’s approach recognizes that aberrant environmental conditions, that enlist incompatible processes, can sometimes cause a kind of mis-behavior to emerge. For example, when a pigeon experiences a colored light paired with grain, conditioning brings about approach to the light. If the light is then presented at a distance from the grain, the pigeon will approach the light even though this has the mal-adaptive consequence of lessening access to grain (Jenkins, 1973). While both Wolpaw and Timberlake assume systems are designed to yield adaptive outcomes, only the behavior systems view recognizes that is not always the case.

## Noxious stimulation can sensitize nociceptive fibers in the spinal cord

The prototype of a spinal reflex is the withdrawal response elicited by the application of a noxious stimulus applied to the distal region of an extremity, the *nociceptive withdrawal response* (Ladle et al., 2007). The classic view of this behavior is that it reflects an innate response, that is wired early in development by genetic factors. At a coarse level, this appears to be true, with the expression of trophic factors within the spinal cord guiding the innervation of sensory fibers, so that they connect to the interneurons needed to drive an adaptive withdrawal response (Granmo et al., 2008). However, this early pattern of innervation reflects a crude/floating somatotopic map, encompassing a diffuse array of connections that has the potential to drive multiple muscles. During early postnatal development (P8-14), the termination pattern is tuned by spontaneous motor activity. This can emerge because spontaneous motor activity produces sensory signals (from skin deformation) that are paired in a Hebbian manner, enabling the selective strengthening of particular sensory-motor connections, a phenomenon known as *somatosensory imprinting* (Petersson et al., 2003; Waldenstrom et al., 2003; Schouenborg, 2008). Interestingly, this tuning can be prevented by pretreatment with a drug that blocks the NMDA receptor (Granmo et al., 2008). Further, in the absence of descending fibers, the tuning is not maintained. Supporting this, a thoracic transection can both prevent and eliminate somatosensory imprinting, increasing the likelihood that a noxious stimulus will elicit an inappropriate approach rather than withdrawal (Schouenborg et al., 1992; Levinsson et al., 1999).

While behavioral studies had shown that stimulus exposure can impact the vigor of a spinal nociceptive reflex, this phenomenon was not extensively studied until the 1990s, when it was recognized that the sensitization of nociceptive pathways in the spinal cord may contribute to the development of chronic pain (Woolf, 1983; Woolf and Thompson, 1991; Willis, 2001; Latremoliere and Woolf, 2009). Nociceptive sensitization develops in response to inflammation or peripheral injury and can bring about an increase in the magnitude of perceived pain (*hyperalgesia*). In addition, there is often an accompanying transformation in the perception of mechanical stimulation, causing a light touch to elicit pain (*allodynia*). These phenomena can be studied in an animal model by applying an irritant (e.g., capsaicin) to one hind paw. To assess the development of an allodynic-like response, plastic monofilaments that vary in thickness/force (von Frey stimuli) are applied to the planter surface of the paw and the stimulus force that engages a withdrawal response is recorded. What is typically found is that treatment with capsaicin enhances reactivity to mechanical stimulation, causing animals to exhibit a withdrawal response to filaments that induce a weak deformation of the skin, below the threshold for engaging nociceptive fibers. Importantly, the amplification of reflexive withdrawal is often accompanied by an enhancement in brain-dependent measures of pain [e.g., a stimulus-elicited vocalization or aversion to an environment (context) that has been paired with mechanical stimulation] (Huang and Grau, 2018). What is remarkable is that the amplification of mechanical reactivity, as measured by a withdrawal response to non-noxious stimulation, is observed in animals that have undergone a rostral spinal transection (Huang et al., 2016), implying that the alteration is due, at least in part, to an intra-spinal modification. Notice here that a change in pain perception arises due to a phenotypic shift in sensory function, that causes signals that normally generate mechanical sensations to elicit pain (Neumann et al., 1996). Contrary to what is sometimes assumed, afferent sensory function is not fixed.

The idea that modifications outside the brain can impact pain processing is supported by electrophysiological studies. Early work had shown that electrical stimulation of sensory fibers at an intensity that engages unmyelinated nociceptive (C) fibers causes a progressive increase in the duration of discharge that fades over the course of minutes (windup; Mendell and Wall, 1965). Subsequent research revealed that a prolonged activation of C-fibers, induced by the application of the TRPV1 agonist capsaicin, inflammation, or nerve injury, can induce a lasting increase in neural excitability (central sensitization) within the spinal cord dorsal horn (Woolf, 1983; Woolf and Thompson, 1991; Willis, 2001; Latremoliere and Woolf, 2009). Subsequent cellular work linked the modification of nociceptive circuits in the dorsal horn to neurochemical systems analogous to those known to underlie learning and memory in the brain (Ji et al., 2003; Figure 3). For example, inducing a lasting modification depends upon the NMDA receptor and an increase in AMPA receptor-mediated excitation. At a cellular level, the neural over-excitation is accompanied by enhanced expression of the immediate early gene *c-fos* and the activation (phosphorylation) of extracellular signal-regulated kinase (ERK). And like many examples of brain-dependent synaptic plasticity, the development of nociceptive sensitization is regulated by BDNF (Pezet et al., 2002; Merighi et al., 2008; Smith, 2014; Huang et al., 2017). Further parallels have been identified by Sandkuhler and his colleagues, who showed that electrical stimulation of nociceptive fibers can induce a form of long-term potentiation (LTP), and that this effect too is blocked by pretreatment with an NMDAR receptor antagonist (Liu and Sandkuhler, 1997; Liu et al., 1998; Sandkuhler and Liu, 1998; Sandkuhler, 2000).

Further work has shown that neural excitability within the dorsal horn is regulated by serotonergic fibers that descend through the dorsolateral funiculus (DLF), which dampen neural excitability by engaging the 5HT-1A receptor, inhibiting the development of nociceptive sensitization and spinally-mediated LTP (Gjerstad et al., 1996; Liu and Sandkuhler, 1997; Sandkuhler and Liu, 1998; Crown and Grau, 2005). Supporting this, bilaterally cutting fibers in the DLF at the thoracic level fosters the development of enhanced mechanical reactivity after capsaicin treatment and increases the expression of cellular indices of nociceptive sensitization in the dorsal horn (e.g., c-fos and pERK; Ji et al., 1999, 2003; Latremoliere and Woolf, 2009). Clinically, the observations imply that a SCI that damages these descending fibers would foster nociceptive sensitization and the development of chronic pain.

More recent work has revealed that SCI enables the development of nociceptive sensitization within the spinal cord by reducing GABAergic inhibition (Huang et al., 2016). As noted above, SCI reduces the expression of the co-transporter KCC2 caudal to injury. This reduces the intracellular Cl^−^ concentration, which attenuates the hyperpolarizing (inhibitory) effect of GABA, removing a brake on neural activity that fosters neural excitation. This alteration in GABA function can be countered by pharmacological treatments that lower the intracellular concentration of Cl^−^and by application of drugs that engage the 5HT-1A receptor, which up-regulates the expression of KCC2 (Huang and Grau, 2018). Likewise, as noted above, training and exercise can up-regulate KCC2 expression, which counters the development of chronic pain after SCI (Cote et al., 2014; Tashiro et al., 2015).

Interestingly, in the absence of SCI, local inflammation within the spinal cord can also induce a depolarizing shift in GABA that fosters nociceptive processing. This effect appears linked to the activation of microglia and the release of BDNF, which reduces KCC2 expression in uninjured animals (Coull et al., 2005; Lu et al., 2009; Beggs and Salter, 2013). Here the effect of BDNF is opposite to what has been reported after SCI, where BDNF has been shown to increase KCC2 expression caudal to injury and counter the development of nociceptive sensitization (Huang et al., 2017). These divergent effects have been linked to the activation of the TrkB receptor by BDNF and the downstream engagement of Shc, which can impact KCC2 expression in opposite ways depending on levels of phospholipase C-γ (PLC-γ; Rivera et al., 2004, 2005). When PLC-γ is present, Shc down-regulates KCC2. However, in the absence of PLC-γ, engaging Shc increases KCC2 expression. Because PLC-γ levels are high in uninjured adult animals, BDNF-induced Shc signaling will cause a reduction in KCC2 expression, bringing an increase in neural excitability that would foster nociceptive sensitization. SCI reduces PLC-γ, which would transform how BDNF affects KCC2 expression. Now, engaging Shc signaling would up-regulate KCC2 expression, re-establishing GABA-dependent inhibition and quelling neural excitation. Interestingly, locomotor training may re-establish GABAergic inhibition because it increases the expression of PLC-γ (Tashiro et al., 2015).

Just as those studying the brain have often assumed that systems within the spinal cord are fixed, those exploring spinal cord plasticity have sometimes assumed sensory fibers behave in a mechanical manner, with the afferent input reliably tied to the extent of injury. Recent findings suggest that this view too needs to be updated—that nociceptive sensitization after SCI may be attributable, in part, to the sensitization of afferent nociceptive neurons (Yang et al., 2014; Walters et al., 2023). As noted above, the cell bodies of afferent neurons are contained within the DRG, which lie proximal to the spinal cord tissue within the epidural space. Under natural conditions, the sensory fibers designed to detect tissue damage/injury would only be engaged by peripheral events—because damage to the spinal cord would be lethal. SCI sets up a non-natural situation wherein the central projections innervate damaged tissue, which can engage retrograde signals that activate the sensory neuron, causing these neurons to exhibit on-going spontaneous activity (at about 1 Hz). This aberrant activity could drive pain circuits in the spinal cord in the absence of peripheral damage, to foster neuropathic pain (Bedi et al., 2010; Yang et al., 2014; Walters, 2018; Walters et al., 2023). The activity could also fuel the development of LTP, amplifying the elicited response. These changes have been shown to be persistent, with on-going activity observed in TRPV1 sensitive neurons weeks after SCI. Further, because the extracellular signals related to injury are diffusely distributed, aberrant activity may arise in adjoining regions, fostering both above-level and below-level pain. Support for this general view comes from studies demonstrating that the development of spontaneous activity within DRG nociceptive neurons is correlated with behavioral indices of neuropathic pain (Bedi et al., 2010). More importantly, silencing a voltage gated Na^+^ channel (Nav1.8) that is exclusively expressed on nociceptive afferents attenuates both the development of spontaneous activity and behavioral signs of neuropathic pain after SCI (Yang et al., 2014).

## Neurons within the spinal cord are sensitive to stimulus–stimulus relations

The findings reviewed above show that engaging nociceptive fibers can sensitize neural excitability within the spinal cord, a modification that enhances behavioral reactivity and pain signaling. Because noxious stimulation has a lasting effect, and is attributable to a single event, it constitutes an example of single stimulus (non-associative) learning (Grau, 2014; Grau et al., 2020). Bolstered by data demonstrating that this effect is mediated by signal pathways implicated in brain-dependent memory (Ji et al., 2003), the phenomenon is widely accepted and recognized to have implications for the treatment of chronic pain (Latremoliere and Woolf, 2009). What has proven more controversial is whether the spinal cord can encode an environmental relation, either between two stimulus events (*Pavlovian conditioning*) or a response and an outcome (*instrumental conditioning*). As we will see, this controversy arose in large measure because learning has been historically couched in associative terms, a process most assume requires a brain (Grau et al., 2022).

**Figure 4:**
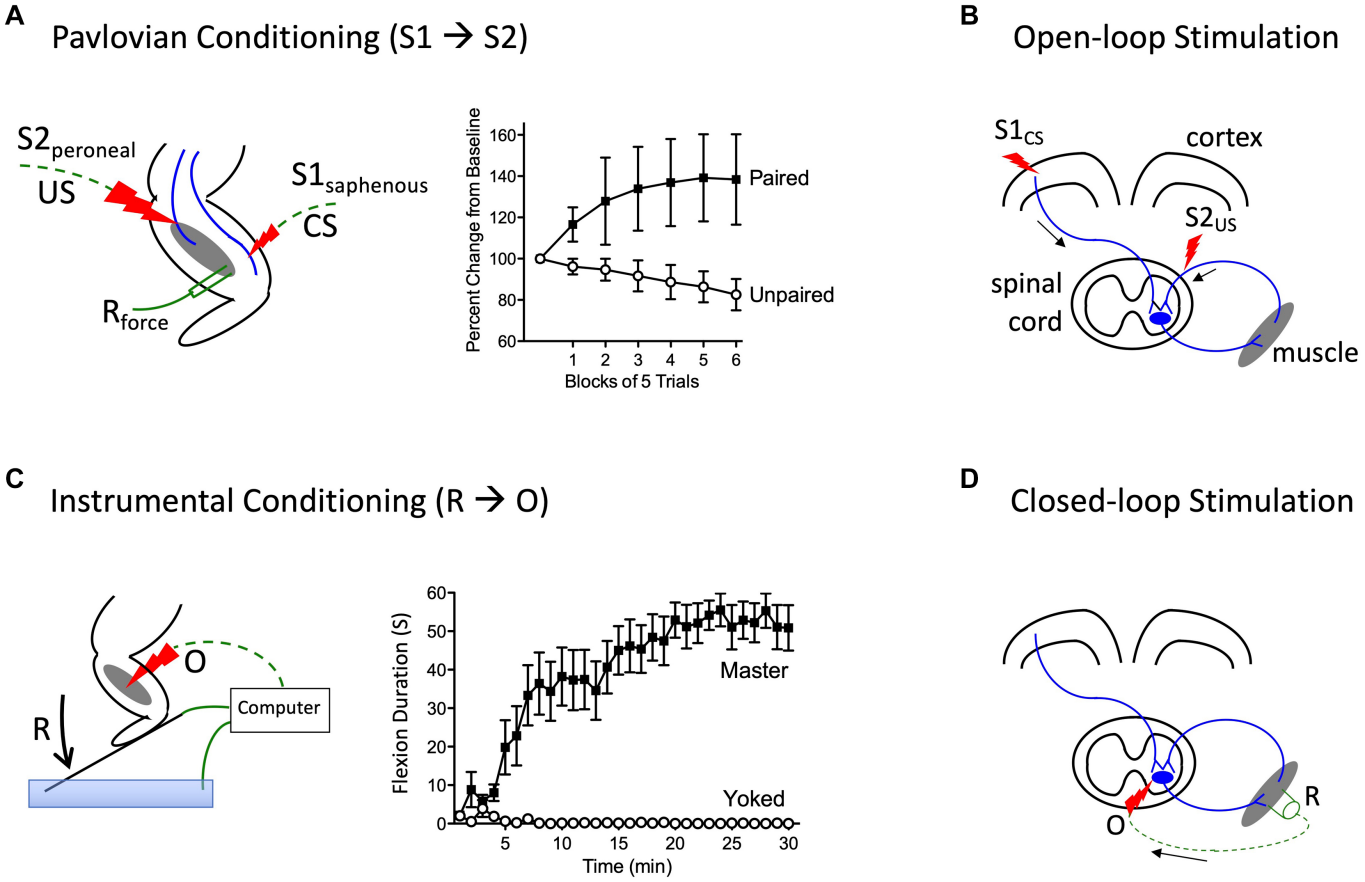
Methods for instituting a Pavlovian (S1→S2) and instrumental (R→O) relation. **(A)** In rats that have received a rostral (T2) transection, pairing electrical stimulation of the peroneal nerve [S2; the unconditioned stimulus (US)] with weak stimulation of the saphenous nerve [S1; the conditioned stimulus (CS)] amplifies the response elicited by S1 relative to animals that experience S1 and S2 in an unpaired manner (Durkovic, 2001). **(B)** Electrical stimulation of the motor cortex (S1) can engage surviving descending (corticospinal) fibers after SCI. Pairing S1 with epidural stimulation, which engages sensory afferents, in a Pavlovian manner (open-loop stimulation) enhances motor performance after SCI (Harel and Carmel, 2016). **(C)** Spinally transected rats (Master) that receive noxious electrical stimulation of the tibialis anterior muscle [the outcome (O)] whenever the leg is extended [the response (R)] exhibit a progressive increase in flexion duration that reduces net exposure to the noxious stimulus. Animals that receive stimulation independent of leg position (Yoked) do not exhibit a change in flexion duration (Grau et al., 1998). **(D)** An instrumental (R-O) relation can also be established using electrophysiological methods (closed-loop stimulation). For example, after SCI, surviving corticospinal neurons can evoke a small evoked (electrical) muscular response (the R). Stimulating the motor neurons (the O) when a R is detected can strengthen motor performance after SCI (McPherson et al., 2015). Error bars indicate the standard error of the mean.

Prior to initiating his classic studies detailing the role of the cerebellum in learning (Thompson, 1986), the neurobiologist Richard Thompson and his students explored whether neural processes within the spinal cord could support a simple form of Pavlovian conditioning (Thompson, 2001). With P. Groves, Thompson had previously detailed the circumstances under which stimulation causes a spinal reflex to decline (habituate) or grow stronger (sensitize), laying the foundation for the dual process model of these phenomena (Groves et al., 1969; Groves and Thompson, 1970; Patterson, 2001a; Thompson, 2001). To examine whether spinal neurons are sensitive to stimulus–stimulus (S-S) relations, stimuli were applied below the waist in animals that had undergone a thoracic transection. Weak stimulation to the saphenous nerve was used for the to-be-trained cue [the conditioned stimulus (CS)], which initially generated a weak flexion response (Figure 4A). This CS was paired with more intense stimulation of the peroneal nerve, which generated a robust unconditioned (unlearned) flexion response prior to training and served as the unconditioned stimulus (US). They found that pairing the events endowed the CS with the capacity to elicit a stronger flexion response [the conditioned response (CR)], relative to animals that experienced the CS and US in an explicitly unpaired manner. Importantly, the training had a lasting effect and group differences were evident when animals were tested under common conditions, demonstrating that mechanisms caudal to the spinal transection are sensitive to S-S relations. Further work showed that learning depends upon the temporal order in which the stimuli were presented, with a forward CS→US relation yielding learning while a backward (US→CS) relation did not, and that presentation of the trained CS alone causes the learned response (CR) to wane (extinction; Durkovic, 2001). And here too, pretreatment with a NMDA receptor antagonist disrupts learning (Durkovic and Prokowich, 1998).

Subsequent studies showed that introducing a S-S relation also affects how spinal neurons process nociceptive signals. In uninjured animals, a cue (the CS+) that has been paired with a noxious shock (the US) produces an inhibition of nociceptive processing (an antinociception) relative to a cue (the CS-) that was never paired with the US (Fanselow, 1986). In intact animals, this conditioned antinociception is mediated by brain processes, which can inhibit nociceptive processing within the spinal cord through descending pathways (McNally et al., 2011). This conditioned antinociception is often assessed by recording the latency to exhibit a spinal nociceptive reflex, tail withdrawal from a thermal stimulus (the tail-flick test). Using this test, we examined whether a conditioned antinociception could be established without input from the brain, in rats that had undergone a thoracic (T2) transection (Joynes and Grau, 1996). Weak stimulation to one hind leg, at an intensity that induced a moderate antinociception, served as the CS and was paired with an intense tail-shock (the US). After 30 trials of training, the paired CS (CS+) elicited antinociception relative to a cue that was presented an equal number of times in an explicitly unpaired manner (the CS−), providing further evidence that neurons within the spinal cord are sensitive to S-S (Pavlovian) relations.

We then went on to explore whether the system could support a number of phenomena traditionally accounted for in terms of attention. For example, it is known that pre-exposure to the CS alone prior to training undermines the development of a conditioned response, a phenomenon known as *latent inhibition* (Lubow, 1973). Likewise, when animals experience a stimulus compound composed of cues that differ in noticeability (salience), learning about the more salient cue typically *overshadows* learning about the weaker stimulus (Pavlov, 1927). We found that presenting a CS alone prior to training, or in compound with a more salient cue, attenuated conditioning in spinally transected rats, providing evidence for both latent inhibition and overshadowing (Grau et al., 1990).

More recent work has used a form of stimulus–stimulus learning to promote motor performance after SCI by pairing epidural stimulation with activity in descending motor pathways (Figure 4B). In rats this can be achieved by applying electrical stimulation over the cervical dorsal root entry zone at an intensity that is subthreshold for eliciting a forelimb response (Mishra et al., 2017). Descending fibers can be engaged by electrically stimulating the cortex at a site that elicits a motor evoked potential (MEP) within the bicep. Instituting this S-S (Pavlovian) relation, which engineers refer to as *open loop* stimulation, amplifies the MEP. Importantly, the effect becomes stronger with repeated pairing and has a lasting effect. It was posited that pairing mattered because it engages a form of *spike-timing dependent plasticity* within the spinal cord (Dan and Poo, 2004). An analogous effect has been induced in humans by activating descending fibers in the corticospinal pathway using transcranial magnetic stimulation (TMS) to engage the cortical region that innervates the leg (Urbin et al., 2017). When TMS was paired with activity in the common peroneal nerve, it amplified the MEP elicited by cortical stimulation. Interestingly, evidence suggests that this example of S-S learning also depends upon a form of NMDAR-mediated plasticity (Donges et al., 2018).

## Neurons in the spinal cord are sensitive to response-outcome relations

Other studies have provided evidence that neural systems within the spinal cord are sensitive to response-outcome (R-O) relations (Grau et al., 1998). This was shown using rats that had undergone a thoracic (T2) transection. Electrical stimulation (shock) was then applied to the tibialis anterior muscle at an intensity that elicited a flexion response (the R). Animals in one group (master) received shock (the O) whenever the leg was extended (Figure 4C). Animals in a second group were experimentally coupled (yoked) to rats in the master condition and received stimulation at the same time, but unrelated to limb position (uncontrollable shock). Application of *response-contingent* (controllable) shock to master rats caused a gradual increase in flexion duration. Animals in the yoked condition exhibited a mechanical response to shock, but did not exhibit an increase in response duration—the index of learning. Importantly, training with controllable shock induced a lasting increase in flexion duration that was evident when animals were tested under common conditions. Further analysis revealed that the change in flexion duration was reinforced by the onset of shock, not its offset (Grau et al., 1998).

The key difference between the master and yoked animals is that the former receives shock when the leg reaches a particular position. The fact that only response contingent shock produces a change in response duration implies that the consequence of shock is modulated by cues related to limb position—proprioceptive cues that indicate either the leg angle (muscle length) or a vector that describes the momentary change in limb position at the time of shock onset (Grau et al., 2012). In either case, learning (the increase in response duration) emerges when the noxious stimulus occurs in a regular (the same) proprioceptive context. As we have noted elsewhere (Grau et al., 2012, 2022), an implication of this analysis is that a response-outcome relation (limb position at the time of shock onset) can be inferred from sensory cues, allowing the organism to *directly perceive* the relation between proprioceptive cues indicative of body location (the response) and the onset of noxious stimulation (the outcome; Gibson, 1979). To appreciate this, consider the feedback associated with tapping one’s finger against a table. The outcome (mechanical feedback related to hitting the table) occurs in a regular proprioceptive context (the downward movement of the finger), allowing the immediate perception of the relation. This account contrasts with a more cognitive view that presumes that the events (the R and the O) that underlie instrumental learning are independently transmitted to the brain, which then derives the underlying (R-O) relation.

At a neurochemical level, spinally-mediated instrumental learning depends upon a form of NMDAR-mediated plasticity, which is modulated by BDNF (Allen et al., 2002; Joynes et al., 2004; Gomez-Pinilla et al., 2007). Further, the strength of the learned response is positively correlated with cellular indices of synaptic plasticity (e.g., CaMKII, CREB, and synapsin I expression).

Above, we described how a form of Pavlovian conditioning (open-loop stimulation) can be used to promote rehabilitation after SCI. An alternative procedure (*closed-loop stimulation*) builds on a form of instrumental conditioning by instituting a R-O relation (Figure 4D). For example, McPherson assessed whether this type of training would benefit recovery of forelimb function in rats that had received a cervical injury (McPherson et al., 2015). A tractable R was obtained by monitoring electromyographic (EMG) activity within a muscle of the impaired limb. When EMG activity (the R) was detected, an electrical pulse (the O) was applied to the cervical spinal cord at a site that drove motor behavior. This R-O training fostered behavioral recovery and had a lasting effect that was evident weeks after training was terminated. Again, the learning was related to a form of spike-timing-dependent plasticity that fostered synaptic connectivity between surviving corticospinal fibers and motoneurons.

## A neurofunctionalist account of learning

Evidence that neural systems within the spinal cord can encode environmental relations was met by researchers within the field of learning with some skepticism, forcing those studying spinal cord plasticity to lay out the defining criteria for learning and address alternative interpretations of the results (Joynes and Grau, 1996; Grau et al., 1998; Grau and Joynes, 2001; Grau, 2014). Two issues proved central: (1) does the experience (training) have a lasting effect; and (2) are the consequences of training evident when animals are tested under common conditions? For both Pavlovian and instrumental learning, these criteria have been met (Grau, 2014; Grau et al., 2020, 2022).

Those seeking to preserve a brain-centric view of learning may acknowledge spinal cord systems are sensitive to environmental relations, but deny that this reflects a form of associative learning, suggesting instead that the learning involves a modification of a pre-existing response tendency rather than a *de novo* association (Grau et al., 2022). The implicit claim is that *true learning* is associative in nature. From this perspective, simple invertebrates and neurons in the spinal cord may be sensitive to Pavlovian and instrumental relations, but this learning depends upon simpler processes that are built upon pre-existing response tendencies. The conclusion is that these examples of learning do not represent a challenge to the traditional view that associative learning requires a brain.

While there are a number of issues lurking here, the core complaint is tied to the formation of a *de novo* link (Gormezano and Kehoe, 1975). From this view, associative learning enables organisms to build a storehouse of knowledge encoding new environmental relations—to build a model of the world. To study this process, researchers have sought paradigms wherein the events have no pre-existing tendency to elicit the to-be-trained behavior. For example, an auditory cue (a tone) may be paired with an air-puff to one eye, establishing a conditioned response (eyeblink) to the tone. Here it is suggested that the tone had no discernable behavioral effect prior to training, implying the learning involved the formation of a new link. As detailed elsewhere (Grau et al., 2022), a problem with this approach is that further probing routinely reveals that the presumably “neutral” CS has some capacity to elicit the to-be-trained response. Indeed, current neurobiological accounts of eyeblink conditioning, the prototype of *associative* learning, assume that the CS-US link is biologically prepared (by a pre-existing connection within the cerebellum; Thompson, 1986).

Likewise, while learning to press a bar (the R) for food (the O) may appear an arbitrary relation for a rat, further analysis has revealed that this example of instrumental learning is built upon pre-existing response tendencies (Timberlake and Lucas, 1989; Timberlake, 1990). Observations such as these suggest that the ideals of associative learning may be seldom achieved in studies of animal learning. Of course, there is considerable variation in the extent to which biological preparedness constrains learning and it is true that learning within the spinal cord is highly prepared. Conversely, forms of learning mediated by the hippocampus, which can encode relations across gaps in time, a spatial map, and what, when, and where an event occurred (episodic memory), are much less constrained. But none of this necessarily implies a qualitative change in the underlying processes. Indeed, at a neurochemical level, commonality appears the rule (Ji et al., 2003; Latremoliere and Woolf, 2009).

**Figure 5:**
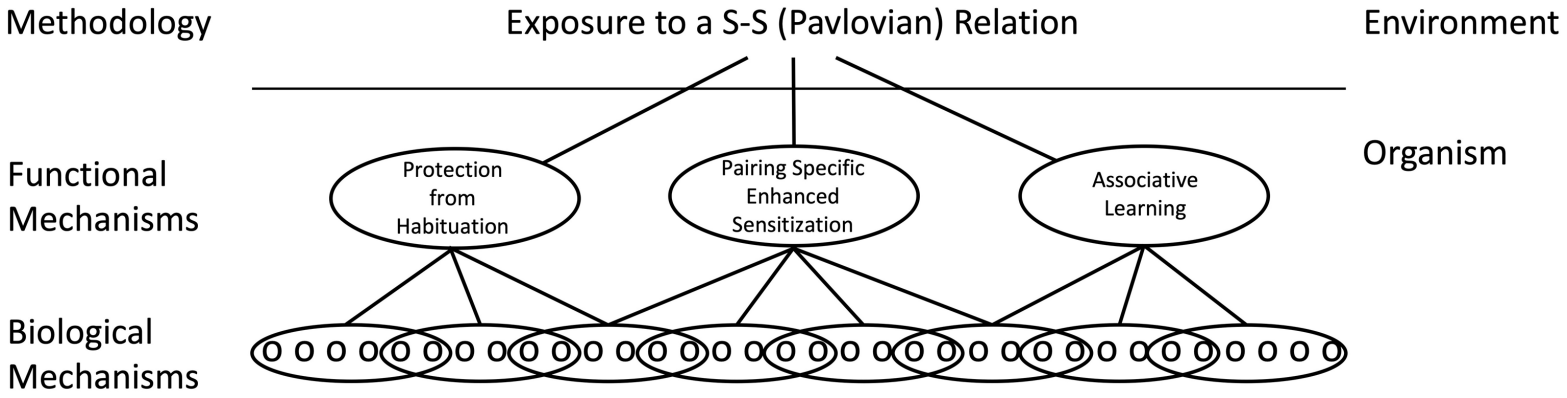
A neural-functionalist perspective on Pavlovian conditioning. It is assumed that environmental relations can be encoded by multiple mechanisms within the organism, which can be distinguished by their functional properties. It is likewise assumed that a functional mechanism can be neurally encoded in multiple ways and that a particular biological mechanism (e.g., NMDA receptor-mediated plasticity) can be enlisted by multiple processes. Adapted from Grau and Joynes (2005a).

While common neurobiological processes may be involved, how the consequent circuits support learning can differ. The assumption here is that the same environmental puzzle (e.g., encoding a stimulus–stimulus relation) may be solved in multiple ways, by systems that have distinct functional properties (Grau and Joynes, 2005a,b). For example, pairing a CS with a US can alter a CS elicited response by slowing the rate of habituation to the CS (protection from habituation), enhancing a pre-existing CS-elicited response (pairing-specific enhanced sensitization), or build upon a new neuronal connection (associative learning; Figure 5). While each mechanism may be governed by some common rules (e.g., a dependence on contiguity, stimulus competition), the underlying processes can be distinguished at a functional level (e.g., by comparing the magnitude of the CR elicited by the trained CS to a cue that is novel). Likewise, there is considerable evidence that R-O relations can be encoded in multiple ways, with learning in some situations reflecting the modification of a pre-existing stimulus–response (S-R) habit and in others a goal directed response that depends upon the current value of the outcome (Domjan, 2015). We have suggested that this diversity in process is best handled by first recognizing that Pavlovian and instrumental conditioning reference the environmental relations that support the learning and that, at a functional level, these relations can be encoded in multiple ways. Here it is assumed that no process is superior to the rest, a view that runs counter to the notion that *true* learning is associative in nature. At a neurobiological level, the processes may often share common elements, but their relative contribution and how they are assembled is assumed to vary. In some cases, the development of a CR may be largely accounted for by an increase in transmitter release from the presynaptic neuron whereas in others, an enhancement in the post-synaptic response could underlie the learning. By identifying how the CNS encodes the events at a functional level, we gain additional insight into how the process operates. By recognizing that the same relation can be encoded in multiple ways, this *neurofunctionalist* approach embraces the diversity of biological solutions (Grau and Joynes, 2005a,b).

## Learning can induce a peripheral memory

We noted above that early work on nociceptive sensitization focused on the enhancement of neural excitability within the dorsal horn and that more recent work has challenged this view by showing that peripheral alterations within the DRG contribute to the increase in neural excitability. Likewise, new findings suggest that researchers may have underestimated the peripheral contribution to some examples of motor learning. Here, the usual assumption was that training alters the efferent motor output from neurons in the ventral dorsal horn. From this view, the application of response-contingent (controllable) shock to a hind leg of a spinally transected rat produces an increase in flexion duration because it increases the efferent drive from motor neurons. Here it was implicitly assumed that peripheral changes contribute little to the behavioral modification. This fits with the general assumption that the elicitation of a muscle response at the NMJ is over-determined, to assure a behavioral response is reliably triggered given motoneuron activity. Building on these assumptions, we sought to identify the intraspinal processes that maintain a prolonged flexion (Hoy et al., 2020). Preliminary work revealed that the application of drugs targeting signal pathways implicated in memory had surprisingly little effect. Given this, we decided to verify our method for applying a drug to the spinal cord through an intrathecal (i.t.) catheter was effective. To confirm this, we administered an anesthetic, the Na^+^ channel blocker lidocaine. We had previously shown that pretreatment with lidocaine blocks the acquisition of a spinally-mediated instrumental response (Crown et al., 2002a), which is not surprising given the drug disrupts the performance of a spinal reflex (e.g., tail withdrawal from radiant heat) within minutes of application (Hoy et al., 2020). But when the drug was applied after 30 min of instrumental training, it had no discernable effect on the maintenance of the behavioral response. Likewise, cutting efferent fibers to the muscle, by transecting the sciatic nerve, blocked learning but not the maintenance of the behavioral response. Even removing the region of the spinal cord between L3 and S3, which has been shown to mediate instrumental learning (Liu et al., 2005), had no effect on the maintenance of the behavioral response. Together, the results suggested that motor output from the spinal cord contributed little to the maintenance of the flexion response.

Neurochemical transmission at the NMJ depends upon acetylcholine (ACh; Sanes and Lichtman, 1999, 2001). To verify that the maintenance of the behavioral response depended upon ACh release, rather than a tonic intramuscular process (latch) that maintained contraction, spinally transected rats were trained for 30 min and then the ACh receptor antagonist curare was applied to the muscle (Hoy et al., 2020). Curare caused the behavioral response to quickly wane, implying a dependence upon ACh release. Further work showed that the learning increased the evoked electrical (electromyography [EMG]) response within the tibialis anterior and that this effect survived a sciatic cut. Confocal microscopy revealed that training increased fluorescent labeling of the ACh receptor, implying an up-regulation that would amplify the elicited response.

We posited that efferent motoneuron output during training, in conjunction with electrical stimulation of the muscle, may strengthen synaptic efficacy at the NMJ in a Hebbian (pairing based) manner. Supporting this, paired stimulation of the efferent nerve and muscle induced an increase in flexion duration without input from the spinal cord (Hoy et al., 2020). Other work suggests that the release of glutamate may also contribute to depolarization at the NMJ. Using immunohistochemical techniques, both vesicular glutamate transporters and the NMDAR have been shown to be present at the NMJ in adult vertebrate skeletal muscles (Mays et al., 2009; Malomouzh et al., 2011). Given this, we examined the effect of applying the NMDAR antagonist MK-801 to the muscle. We found that the drug disrupted both the acquisition and the maintenance of the behavioral response, implying that NMDAR-mediated plasticity plays a role (Hoy et al., 2020).

These results are consistent with a growing body of work that over-turns some long held views regarding NMJ function in adult vertebrates. One is that muscle memory is a myth—that training does not affect the strength of the synaptic connection at the NMJ, which is designed to function well above threshold to assure a muscular response is reliably elicited. While this may be generally true, it does not mean that plastic potential disappears after the system matures. Prolonged execution of a specific response can increase synaptic efficacy enabling contraction with lower transmitter release. In many regards, this conclusion is not surprising, given that the selection of NMJ’s during development depends upon a competitive process linked to coordinated activity (Personius and Balice-Gordon, 2000). Secondly, the work calls into question the standard view of neurochemical communication at the NMJ in a mature vertebrate, which was assumed to depend upon ACh alone. Early in development, and in invertebrates, glutamate plays a pivotal role at the NMJ (Personius et al., 2016). Given this, it should not be surprising that glutamate continues to play a functional role in adult vertebrates.

## Engaging plasticity impacts plastic potential (metaplasticity) within the spinal cord

To demonstrate learning, it is important to show that training has a lasting effect, that is evident when animals are tested under common conditions (Rescorla, 1988). To address this issue in our instrumental learning paradigm, we tested spinally transected rats that had received either controllable (master) or uncontrollable (yoked) stimulation for 30 min with response contingent shock (Grau et al., 1998). We also included a group that had been set-up in the same manner, but never received stimulation (unshocked). We found that animals that had received controllable stimulation re-acquired the behavioral response faster than the naïve group, demonstrating a savings effect indicative of learning. Our assumption was that the yoked animals would show no evidence of savings and learn at a rate comparable to the previously unshocked group. Contrary to our expectations, animals that had received uncontrollable shock exhibited a shock-elicited flexion, but not an increase in flexion duration—our index of learning. It appears that prior exposure uncontrollable shock induced a learning impairment, an effect reminiscent of the phenomenon of learned helplessness (Maier and Seligman, 2016).

Further work showed that a relatively brief period of uncontrollable stimulation (6 min of intermittent shock provided on a variable schedule) has a lasting effect that blocks learning when animals are tested with response-contingent shock 24 h later (Crown et al., 2002b). Further, the deficit reflects a general effect on plastic potential, impairing the capacity to learn after uncontrollable stimulation is applied to the opposite leg or even the tail. We posited that uncontrollable stimulation might impair learning because it sensitizes nociceptive circuits in the dorsal horn, producing a diffuse state of over-excitation that saturates plasticity. Supporting this, treatments that induce nociceptive sensitization (e.g., peripheral treatment with capsaicin) produce a learning impairment (Ferguson et al., 2006). Further, like capsaicin, uncontrollable shock enhances reactivity to mechanical stimulation applied to the hind paws. This over-excitation has been linked to the expression of the pro-inflammatory cytokine tumor necrosis factor (TNF) and an upregulation of Ca^++^ permeable AMPA receptors (Huie et al., 2012a, 2015). The long-term effect of uncontrollable stimulation depends upon protein synthesis and NMDAR-mediated plasticity (Patton et al., 2004; Ferguson et al., 2006). Interestingly, like the learning impairment observed after uncontrollable stimulation in intact rats, the spinally-mediated deficit is reversed (temporarily) by administration of the opioid antagonist naltrexone (Joynes and Grau, 2004; Washburn et al., 2008). We have also recently discovered that the adverse effect of noxious stimulation is gated by limb position; noxious shock and capsaicin induce a learning impairment if given while the hind legs are extended, but not if the legs are maintained in flexed (protective) position (Hudson et al., 2022). It appears that the proprioceptive context modulates how noxious stimulation affects spinal cord function.

If spinally transected rats are given controllable shock prior to uncontrollable stimulation, no learning impairment is observed (Crown and Grau, 2001). Conversely, administration of controllable shock (in compound with an opioid antagonist) eliminates the learning impairment. Exposure to controllable stimulation also counters the learning impairment and enhanced mechanical reactivity produced by peripheral application of capsaicin (Hook et al., 2008). These restorative effects have been linked to the expression of BDNF (Huie et al., 2012b).

Taken together, the results imply that controllable and uncontrollable stimulation have opposing effects on spinal cord plasticity, the former enables learning while the latter disables it. In both cases, learning affects future plastic potential, a kind of plasticity of plasticity (metaplasticity; Abraham and Bear, 1996; Abraham, 2008; Grau et al., 2014; Grau and Huang, 2018).

## Spinal cord neurons have a sense of time

**Figure 6:**
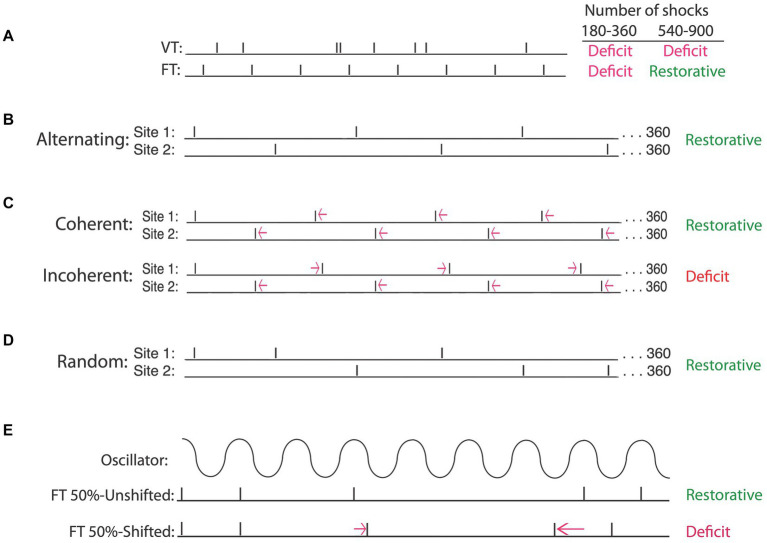
Intermittent stimulation can have distinct effects on spinal cord plasticity depending upon the underlying temporal relation. **(A)** When the interval between stimuli is randomly varied [variable time (VT)], a learning impairment is observed when animals are tested after 180–900 stimuli (Deficit). If stimuli occur in a regular manner [fixed time (FT)], a learning deficit is observed when animals are exposed to 180–360 stimuli. Exposure to additional stimulation (540–900) has a restorative effect that counters the learning deficit. **(B)** A restorative effect emerges when the locus of FT stimulation is alternated across regions of the body (e.g., hind leg and tail). **(C)** Temporally displacing alternating stimuli by a small amount preserves the regularity of stimuli applied at each site. Displacing the stimuli in opposite directions introduces an irregular relation (incoherent) across sites and produces a learning deficit. If the stimuli are displaced in the same direction (coherent), a regular pattern can be abstracted across sites and a restorative effect emerges. Regularity can also be abstracted when the site of stimulation is randomly varied across sites **(D)** and when half of the stimuli are randomly omitted **(E)**, provided the stimuli remain in phase (FT 50%-Unshifted). Shifting the phase relation after a stimulus is omitted (FT 50%-Shifted) disrupts the abstraction of regularity, causing the same number of stimuli to induce a learning deficit. Adapted from Lee et al. (2015), Lee et al. (2016), and Grau et al. (2022).

Having shown that exposure to uncontrollable intermittent stimulation impairs spinal cord plasticity, we sought to identify the circumstances under which this effect develops. When we compared intermittent stimulation to continuous, we found that only the former induced a learning impairment (Crown et al., 2002b). Indeed, concurrent exposure to continuous stimulation has a protective effect that blocks the induction of the learning impairment by intermittent stimulation. Given the stimulation must be intermittent, we then set out to elucidate the stimulus frequency and intensity that has an adverse effect. We found that the deficit emerges at an intensity that engages unmyelinated pain (C) fibers (Baumbauer et al., 2008). To explore the effective frequency range, we modified the computer program used to generate uncontrollable stimulation. Our usual procedure applied brief (100 msec) shocks on a variable time (VT, 0.2–3.8″) schedule, with shocks spaced an average of 2 s apart (0.5 Hz). Recognizing that it would be easier to manipulate stimulus frequency if the interval between the stimuli was fixed, we examined the effect of administering intermittent shock for 6 min (180 shocks) in a regular (fixed time [FT]) or variable time (VT) fashion. As expected, both shock schedules produced a lasting learning impairment (Figure 6A). This made sense given the large literature on timing, which has linked the capacity to discriminate alternative temporal schedules to neural systems in the brain (Mauk and Buonomano, 2004). From this view, there was little reason to expect that neurons within the spinal cord could discriminate FT and VT stimulation.

In a subsequent experiment, we assessed the impact of increasing the duration of stimulus exposure 5-fold, giving animals 900 shocks on either a VT or FT schedule. To our surprise, only VT stimulation induced a learning impairment (Baumbauer et al., 2008, 2009). Given that fewer FT shocks (180) impaired learning, but 900 did not, the results suggested that continued exposure to FT stimulation (540–720 more shocks) has a restorative effect. Further work showed that an extended exposure to FT stimulation blocks the induction of a learning impairment when animals are given VT stimulation 24 h later. The induction of this protective effect was prevented by pharmacological treatments that block protein synthesis or the NMDA receptor. Taken together, the results imply that continued exposure to regular (FT) stimulation has a protective/restorative (metaplastic) effect analogous to that produced by training with controllable stimulation (Baumbauer and Grau, 2011), and here too, the beneficial effect of training was linked to the expression of BDNF (Baumbauer et al., 2009).

What makes these findings especially remarkable is that they imply that the spinal cord can discriminate whether stimuli occur at random or fixed intervals, suggesting that neural systems within the spinal cord can abstract how stimuli are distributed over time. We posited that the capacity to detect the regularity of stimulation may be linked to the engagement of an internal oscillator, possibly the CPG that drives stepping (Baumbauer et al., 2009; Lee et al., 2015, 2016). Consistent with this, the restorative effects of regular stimulation emerge within the frequency range of stepping (de Leon et al., 1994; Cha et al., 2007). If regular stimulation has a special effect because it engages an internal (central) oscillator, it could potentially abstract regularity when stimuli are presented to distinct regions of the body (across sensory dermatomes). Supporting this, we showed that an extended exposure to regular stimulation induces a restorative effect when half of the shocks are applied to the leg while the other half are presented to the tail (Figure 6B), and that this is true independent of whether the locus of stimulation varies in a regular or random manner (Figure 6D).

If an internal oscillator is engaged by regular stimulation, and has some momentum, the system should be able to derive regularity when some of the stimuli are omitted (Figure 6E). As predicted, we found that randomly omitting half the shocks had no effect on the development of restorative effect (Lee et al., 2016). Finally, if an internal oscillator effectively predicts the occurrence of the next shock, based upon a constant period, stimuli would have to remain in phase. As hypothesized, a restorative effect does not emerge if regular stimulation is given across dermatomes at different frequencies (Figure 6C). Likewise, when shocks are randomly omitted, a protective effect only emerges if the shocks remain in phase (Figure 6E).

The fact that randomly omitting half the shocks given on a FT schedule does not affect the emergence of the restorative effect has implications for the conditions that engage this process. As noted earlier, when no shocks are omitted, a restorative effect emerges after 540 shocks—360 is not sufficient. But when 720 shocks are given, and half are omitted, the restorative effect is observed (Lee et al., 2015). This implies that it is not the number of shocks that is critical. Rather, what appears critical is how long the CPG is engaged. This is consistent with other work demonstrating a form of savings across days. Animals given a single bout of 360 FT shocks exhibit a learning impairment. If they receive two bouts of 360 FT shocks, 24 h apart, the capacity for learning is restored. Importantly, the two bouts do not have to be the same frequency. What appears to be summated across days is a marker linked to the duration of regular stimulation, not the specific frequency.

Evidence that the detection of regularity is linked to the CPG that underlies stepping was obtained using a surgical manipulation. Prior work has shown that spinally-mediated instrumental learning depends upon neurons within the lower lumbosacral spinal cord, between L3 and S2 (Figure 1E) (Liu et al., 2005). Interestingly, the neural circuit that mediates the CPG used for stepping appears to lie in a more rostral region, L1-L2 (Cazalets et al., 1995; Magnuson et al., 1999). Given this, we should be able to surgically disconnect the circuit needed for instrumental learning from the CPG by transecting the spinal cord at L3. Minus access to the CPG, FT stimulation should not have a restorative effect, and instead produce a learning impairment, which is what we found (Lee et al., 2016).

The results are consistent with other work demonstrating that regular movement, induced by passively moving the hind limbs over an extended period of time or training animals to step on a treadmill, has a restorative effect (Alluin et al., 2011; Rossignol, 2017). Further, regular stimulation of the perineum, which is often used to encourage stepping on a treadmill, may promote behavior because it engages the CPG. Interestingly, studies examining the consequences of step training have shown that animals trained at one stepping rate exhibit improved performance when tested at different treadmill speeds (Edgerton et al., 1997, 2004). Again, what may be critical is engagement of the CPG for an extended period of time, not the particular frequency used in each bout of training.

Regular stimulation can also impact neuronal function in the cervical spinal cord, which regulates breathing. Mitchell and his colleagues have shown that intermittent bouts of hypoxia can enhance activity in the (phrenic) nerve that drives respiration, inducing a lasting effect [phrenic long-term facilitation (pLTF)] that has been linked to increased expression of BDNF (Baker-Herman et al., 2004; Dale-Nagle et al., 2010; Fields and Mitchell, 2015; Fuller and Mitchell, 2017). While a continuous period of hypoxia can enhance the rate of respiration, it does not induce pLTF or impact BDNF expression. In rats, daily intermittent hypoxia promotes the recovery of breathing capacity after SCI and in combination with ladder walking, promotes the restoration of forelimb function. In humans, daily intermittent hypoxia combined with walking practice increased endurance by 38% (Hayes et al., 2014; Navarrete-Opazo et al., 2017).

Our work on timing within the spinal cord was originally motivated by a simple question—what type of process allows neurons within the spinal cord to discriminate (and provide distinct physiological consequences) whether the stimuli occur in a random or regular (predictable) manner? Our experiments explored whether this might be linked to a kind of neurochemical/physiological hourglass or an internal oscillator (Boulos and Terman, 1980). As we have seen, our results suggest that regularity is tied to an oscillator, which we linked to the CPG that drives the rhythmicity of stepping. What is especially surprising is the system can abstract regularity when stimuli are randomly omitted or when the locus of stimulation is varied. Here the computational capacity of the system goes well beyond an elicited reflex, demonstrating a cognitive-like ability to abstract relations to modulate performance and plastic potential.

## Spinal cord injury removes the GABA-dependent brake on neural excitability (ionic plasticity)

We noted earlier that SCI brings about a depolarizing shift in GABA (ionic plasticity), which removes a brake on neural excitability (Viemari et al., 2011). Evidence suggests that this enables the development of nociceptive sensitization, which we have argued underlies the learning impairment observed after uncontrollable shock (Ferguson et al., 2012). These observations suggest that drug treatments that restore the inhibitory effect of GABA should attenuate both the enhanced mechanical reactivity and learning impairment induced by uncontrollable shock in spinally transected rats, and recent findings are consistent with this prediction (Huang et al., 2016; Grau et al., 2022; Hudson and Grau, 2022). In addition, a depolarizing shift in GABA action, which accompanies spinal cord transection, appears necessary for spinally-mediated instrumental learning. If the hyperpolarizing effect of GABA is re-established in spinally transected rats, by lowering the inward flow of Cl^−^with the NKCC1 blocker bumetanide, spinally transected rats fail to learn. Taken together, the results imply that ionic plasticity enables learning within the spinal cord.

These observations suggest that the adult spinal cord, minus injury or inflammation, may indeed be relatively immutable, with the inhibition action of GABA maintaining circuits laid down early in development (Ben-Ari, 2002, 2014). Some may take this as evidence for the traditional view. We take an alternative position (Grau et al., 2022), because other work has shown that these observations are not unique to the spinal cord (Hudson and Grau, 2022). A depolarizing shift in the action of GABA has been shown to contribute to a variety of brain-dependent pathologies linked to neural over-excitation (e.g., epilepsy, addiction, Rett syndrome). Moreover, dampening the inhibitory effect of GABA may be a prerequisite to plasticity, LTP, and learning within the brain. Across the CNS, GABA may function to maintain neural circuits laid down by development and learning.

## Noxious stimulation impairs recovery and fosters the development of chronic pain after SCI

Given noxious stimulation induces a form of maladaptive plasticity in spinally transected animals, we hypothesized that it could adversely affect recovery after a contusion injury (Grau et al., 2004). This is clinically important because many SCIs are accompanied by other tissue damage (polytrauma). To examine whether pain after SCI affects injury-related processes, rats were given a moderate contusion injury to the lower thoracic spinal cord. The next day, nociceptive fibers were engaged by exposing animals to 6 min of uncontrollable tail-shock or applying capsaicin to one hind paw. Both treatments disrupted long-term behavioral recovery, producing a drop in locomotor performance that was evident 6 weeks later (Grau et al., 2004; Turtle et al., 2018). Noxious stimulation soon after injury (within the first 4 days) also fostered the development of spasticity and increased tissue loss at the site of injury. Importantly, the adverse effect of intermittent shock was only observed if the stimulation was given in an uncontrollable manner; an equal number of controllable shocks had no effect. Further, engaging pain fibers soon after injury fostered the development of chronic pain (Grau et al., 2004; Garraway et al., 2014). Other studies have shown that noxious stimulation during the chronic phase of injury can adversely affect performance, but does not have a lasting effect (Bouffard et al., 2014; Caudle et al., 2015).

A physical blow to the spinal cord produces an immediate (*primary*) injury. As described above, this injury then engages processes that drive cell death and fuel inflammation (Beattie and Bresnahan, 2000), producing a pro-inflammatory storm that expands the area of tissue loss (*secondary* injury). We posited that noxious stimulation after injury has an adverse effect because it fuels secondary processes. To explore this possibility, we collected the injured spinal cord soon after animals received noxious stimulation. We found that nociceptive stimulation amplified the expression of pro-inflammatory cytokines (IL-1, IL-18, TNF) and signals (e.g., caspase 1, 3, 8) that drive cell death (Garraway et al., 2014; Turtle et al., 2018).
In the course of doing these experiments, we noticed that the protein samples were color coded—those from rats that had received noxious stimulation were tinted red (Turtle et al., 2019). Spectrophotometry showed increased absorbance at the wavelength (420 nm) associated with hemoglobin. Cellular assays for hemoglobin confirmed that nociceptive stimulation increased blood content, implying an infiltration of blood (hemorrhage) at the site of injury. Because some blood components are neurotoxic (Regan and Guo, 1998; Losey et al., 2014), this would expand the area of tissue loss.

As noted earlier, descending fibers normally quell nociceptive activity in the spinal cord (Fauss et al., 2021). Given this, we hypothesized that cutting communication with the brain would amplify nociception-induced hemorrhage in contused rats. Contrary to our expectations, the transection had the opposite effect—it blocked nociception-induced hemorrhage (Reynolds et al., 2019). A rostral transection also blocked the up-regulation of pro-inflammatory cytokines and signals indicative of cell death. The surprising implication is that rostral (brain) systems can drive tissue loss after SCI. We presumed that these brain systems were driven by surviving ascending fibers. If this is true, reversibly blocking communication with the brain using lidocaine applied at T2 should prevent nociception-induced hemorrhage, which it did (Davis et al., 2020). Lidocaine treatment also blocked the adverse effect noxious stimulation has on long-term recovery. These observations led us to hypothesize that treatments that diminish brain activity (e.g., general anesthesia) should have a protective effect, and that too is true (Davis et al., 2023). These findings have important clinical implications, suggesting that inhibiting cellular activity within the spinal cord (using a local anesthetic) or inducing a coma-like state (using a general anesthetic) could reduce tissue loss after SCI.

We have shown that engaging sensory fibers that drive a conscious state of pain soon after injury promotes tissue loss and impairs recovery. Given this, we naturally hypothesized that administration of an analgesic, such as morphine, would lessen the adverse effect of noxious stimulation. Surprisingly, administration of morphine at a dose that blocks behavioral reactivity to noxious stimulation does not attenuate secondary tissue loss or the impairment in long-term recovery (Hook et al., 2007, 2009; Turtle et al., 2017). What was especially concerning is that morphine treated rats exhibited greater tissue loss and increased mortality, raising concerns regarding the clinical use of opiate analgesic soon after injury (Hook et al., 2007, 2017).

Our results suggest that the adverse effect of noxious stimulation after injury is due, in part, to a brain-dependent process that fosters the infiltration of blood (hemorrhage) at the site of injury. We assume that this effect depends upon both local (at the site of injury) and systemic processes. Engaging nociceptive (C) sensory fibers can initiate the expression of proinflammatory cytokines at the site of injury and weaken the blood spinal cord barrier (Steinhoff et al., 2014). At the same time, surviving ascending nociceptive fibers could engage a fight-or-flight response that drives a burst of sympathetic activity, bringing a rise in heart rate and blood pressure, Given the weakened state of the blood spinal cord barrier at the site of injury, the rise blood pressure could fuel hemorrhage. Recent work has confirmed that noxious electrical stimulation produces a surge in blood pressure and blood flow (Strain et al., 2021). Further, pharmacologically attenuating the rise in blood pressure, by administering the alpha-1 adrenergic receptor inverse agonist prazosin attenuated the rise in blood pressure, hemorrhage, and the adverse effect noxious stimulation has on long-term recovery. Conversely, pharmacologically inducing a rise in blood pressure, by administering adrenergic agonist norepinephrine a day after injury, impaired recovery and increased tissue loss. These observations are consistent with other work showing that hypertension at the time of injury is associated with a decrement in recovery (Nielson et al., 2015).

The findings outlined above are consistent with other studies demonstrating that SCI can engage systemic processes that can impact tissue loss, recovery, and wellbeing. Of course, SCI recruits both local and systemic components of the immune system, which can have opposing effects on long-term recovery (Popovich, 2014; Schwab et al., 2014); bringing a benefit through the clearance of cellular debris, but limiting re-innervation through the production of a fibrotic (glial) scar (Yang et al., 2020). Beyond this, there is a loss of descending regulatory control over components of the sympathetic nervous system innervated by fiber pathways below the injury (DiSabato et al., 2023). The consequent dysregulation causes systemic immune and metabolic dysfunction that can impact multiple major organ systems, including the liver, lungs, gut, and urinary tract, increasing susceptibility to urinary and lung infections, gut dysbiosis, and a disruption in lipid metabolism (metabolic syndrome; Kopp et al., 2017; Holmes and Blanke, 2019; Kigerl et al., 2020; Rodgers et al., 2022). Pneumonia and urinary tract infections are among the leading causes of mortality after SCI (Schwab et al., 2014; DiSabato et al., 2023; Michel-Flutot et al., 2023). Further, immune dysregulation and an increase in circulating pro-inflammatory cytokines can promote depression and pain (Maier and Watkins, 1998; Slavich and Irwin, 2014; Lees et al., 2015). Finally, these processes can negatively impact tissue sparing and adaptive plasticity at the site of injury. Indeed, preclinical research has shown that liver dysfunction undermines long-term recovery (Failli et al., 2012; Goodus et al., 2021).

## Conclusion

Historically, many have seen the spinal cord as a conduit for neural impulses to/from the brain with a limited capacity to organize some simple reflexive responses. From this perspective, the orchestration of complex behavior, the recognition of response-outcome relations, pain modulation, timing, learning, and memory are the province of the brain. The work we have reviewed supports an alternative position, that recognizes the computational power of neural assemblies within the spinal cord. We align with Windhorst (2007) who argued:

“Those who believed the spinal cord and peripheral motor plant to be well-understood and thus turned their attentions to higher centers of motor planning and coordination (e.g., cerebral cortex and cerebellum) now find that their edifices are built upon ‘the shifting sands of spinal segmental circuitry’.”

As we have seen, neural machinery within the spinal cord can organize coordinated stepping and modify its execution in response to changing environmental demands (e.g., an obstacle; Edgerton et al., 2004; Rossignol and Frigon, 2011; de Leon and Dy, 2017). Noxious stimulation can sensitize nociceptive circuits in the dorsal horn and this effect is mediated by signal pathways analogous to those identified in the study of brain-dependent learning and memory (Sandkuhler, 2000; Ji et al., 2003). At a functional level, neural systems in the spinal cord are sensitive to Pavlovian (stimulus–stimulus) and instrumental (response-outcome) relations and have the capacity to abstract regularity (Grau, 2014; Grau et al., 2022). Further, engaging these processes can influence the capacity to learn, demonstrating a form of metaplasticity (Abraham and Bear, 1996; Abraham, 2008; Grau et al., 2014; Grau and Huang, 2018). And these insights have been shown to impact recovery after SCI (Grau et al., 2004; Edgerton et al., 2008; Garraway et al., 2011, 2014; McPherson et al., 2015; Turtle et al., 2017; Courtine and Sofroniew, 2019; Davis et al., 2020, 2023; Jo and Perez, 2020; Mitchell and Baker, 2022) fueling an optimism that, by harnessing the inherent capacity of the spinal cord, rehabilitation can restore function and counter the development of chronic pain and spasticity.

**Figure 7:**
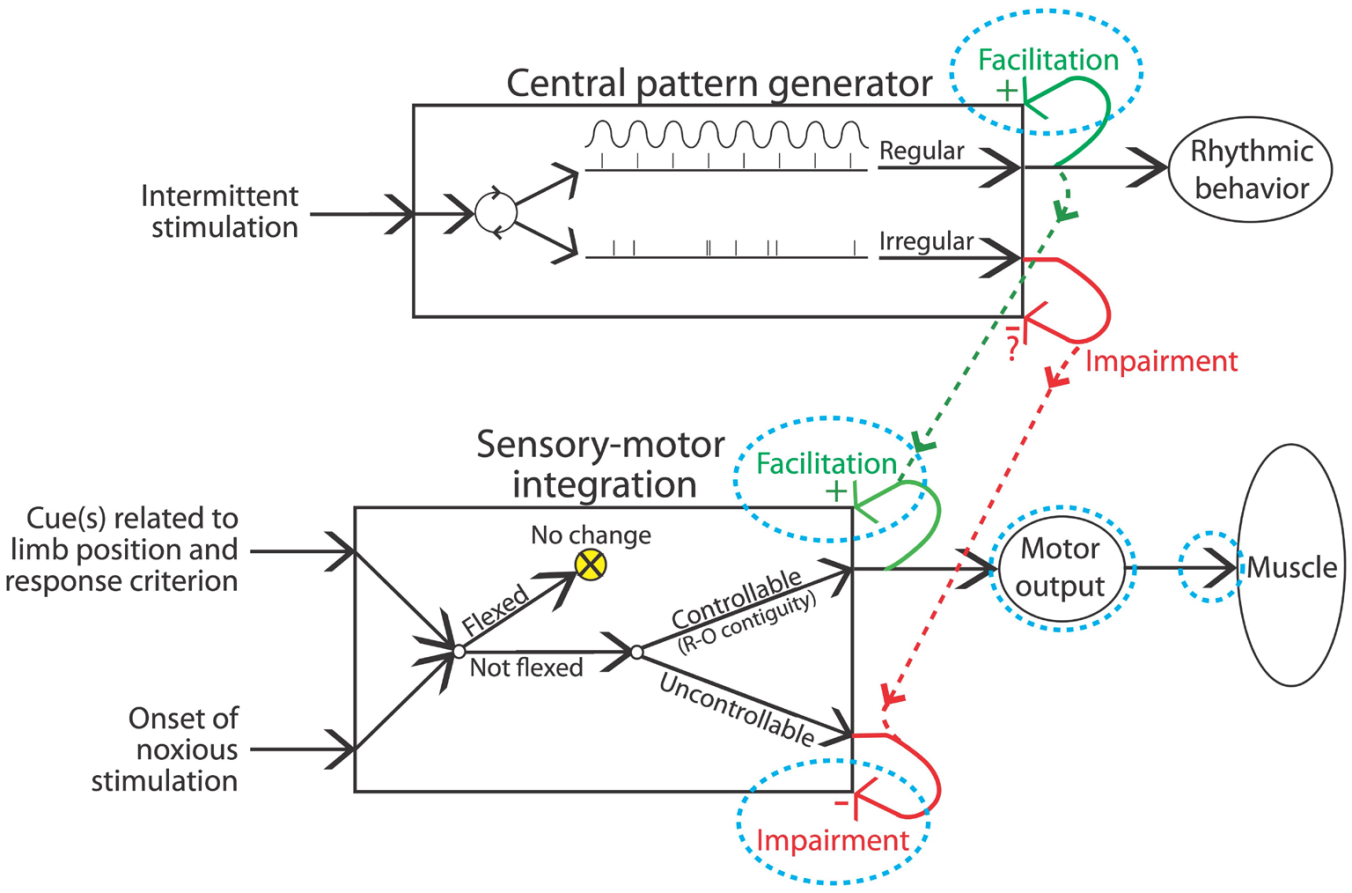
A schematic illustrating the intra-spinal processes that mediate instrumental conditioning, timing, and the consequences of uncontrollable stimulation. Evidence suggests that neural processes within the caudal lumbosacral (L3-S2) spinal cord enable sensory-motor integration (lower box). The effect of noxious stimulation appears to be gated by proprioceptive cues related to limb position; if the leg(s) is flexed, stimulation has no impact on spinal function (blue circle). If the leg is not flexed, a biologically prepared circuit enables the rapid detection of a relationship between the current limb position (the R) and the onset of noxious stimulation (the O). If there is a R-O relation, the stimulation is classified as controllable, which fosters the performance of a motor response that reduces net exposure to noxious stimulation. In the absence of a R-O relation (uncontrollable stimulation), a state of over-excitation is induced that enhances reactivity to mechanical stimulation and induces a lasting impairment in relational learning. Conversely, exposure to controllable stimulation has a restorative effect that fosters learning and counters the adverse effect of uncontrollable stimulation. Other work indicates that a central pattern generator exists in the rostral (L1-L2) spinal cord (upper box) that can be entrained by regular stimulation. Evidence suggests that regularity can be abstracted when stimulation is applied to different regions of the lower body and when some stimuli are randomly omitted. Periods of regular stimulation can foster rhythmic behavior, the abstraction of regularity across days (savings), and counter the adverse effects of uncontrollable stimulation (green lines). Exposure to stimuli that occur in a variable (irregular) manner impairs instrumental learning. Further work is needed to determine whether irregular stimulation also interferes with the abstraction of regularity (red?). Research is also needed to determine how sensory-motor integration impacts the central pattern generator. Evidence suggests that noxious stimulation can interfere with CPG function and the generation of rhythmic behavior (Bouffard et al., 2014; Caudle et al., 2015), implying that the dashed red line reflects a bi-directional process. It is not known whether exposure to controllable stimulation fosters the engagement of the CPG. Note that a ‘+’ and ‘–‘indicate how processes affect function, not the nature of neural communication (i.e., whether an excitatory or inhibitory process underlies the effects). The consequences of training that have been shown to have a lasting effect (implying a form of memory) are enclosed with dashed circles. Adapted from Grau et al. (2022).

Researchers exploring motor performance have long recognized the complexity of spinal circuits, which handle the coordination of motor commands, drive rhythmic behavior, and can adapt to perturbations (Edgerton et al., 2004; Rossignol and Frigon, 2011). From this perspective, the execution of locomotor performance occurs within an organizational structure that is not strictly hierarchical, but instead occurs within an interactive network that enables a form of shared governance [a heterarchy; (McCulloch, 1945; Cohen, 1992)]. Our work suggests that the same is true for the regulation of pain, with nociceptive signals regulated by neural mechanisms within the spinal cord (Figure 7)—a computational system that is capable of abstracting response-outcome and temporal relations (Grau, 2002). Further, experience can have a lasting impact on how these systems operate, to mute or amplify motor responses and the signal relayed to the brain.
Just as brain-centric researchers have underestimated the processing power of the spinal cord, those wedded to the central nervous system have sometimes underestimated the role of peripheral processes. Recent findings show that exposure to a noxious stimulus can induce a state of hyperexcitability in afferent sensory neurons that can foster the development of chronic pain and that prolonged execution of a behavioral response can engage alterations at the NMJ that enhance its efficacy, providing evidence for a kind of muscle memory (Bedi et al., 2010; Hoy et al., 2020; Walters et al., 2023). And we should not forget that, while much has been learned about the capacities of spinal circuits isolated from the brain, those exploring spinal cord systems must take into account how these processes are impacted by, and interact with, brain systems (Wolpaw, 2018; Wolpaw and Kamesar, 2022).
While much of our review has focused on the plastic potential of circuits within the spinal cord, we have acknowledged that GABAergic inhibition will limit neural excitability/plasticity within the uninjured adult spinal cord, a hyperpolarizing effect that we assume helps to maintain circuits laid down early in development. In this way, the adult spinal cord may appear hardwired (Grau et al., 2022). Likewise, we have noted how learning in the spinal cord builds upon pre-existing circuits—that it is biologically prepared by genetic and developmental processes. Here, one might attempt to save the traditional view by arguing the brain is a flexible system, adaptable and unconstrained. We instead suggest the opposite, that GABA-dependent inhibition preserves neural circuits laid down by development and learning throughout the CNS and that learning in both the spinal cord and brain is constrained by our evolutionary past. From this view, the spinal cord is governed by analogous rules and at a neurochemical level, employs the same signal pathways. There are no obvious qualitative differences.
Work over the last 50 years suggests the neural systems within the spinal cord play an integral role in registering, modulating, and elaborating sensory/motor signals. It is basic component of the CNS and can serve as an ideal model system for exploring the processing capacity and limits of neural circuits. And while it is often difficult to link neurobiological modifications in discrete brain regions to behavior, at the level of the spinal cord, just a few synapses may intervene, simplifying the application of our linking hypotheses. Moreover, unpacking how the spinal cord functions often has important clinical implications. Beyond this, those seeking to understand how the brain processes information need to know the types of information contained within the signal from the spinal cord. As we have outlined, relations presumed to be abstracted by the brain may have already been derived by processes within the spinal cord. Conversely, an understanding of how neural circuits within the spinal cord can orchestrate behavioral action will inform our views of the motor commands needed to drive behavior, with evidence suggesting that the structure of behavior is often organized by local circuits.

## Author contributions

JG wrote the first draft of this manuscript, integrating components provided by KH, DJ, and SP. The final draft was edited by JG. All authors contributed to the article and approved the submitted version.
